# Differential gut microbiota composition in β-Thalassemia patients and its correlation with iron overload

**DOI:** 10.1038/s41598-024-75456-4

**Published:** 2024-10-11

**Authors:** Poochit Nonejuie, Alisa Wilantho, Daniel McDonald, Htut Htut Htoo, Jenjira Chalerm, Anupriya Tripathi, Chumpol Ngamphiw, Sissades Tongsima, Rob Knight, Kittiphong Paiboonsukwong, Suthat Fucharoen

**Affiliations:** 1 Institute of Molecular Biosciences, Mahidol University, Nakhon Pathom, Thailand; 2 National Center for Genetic Engineering and Biotechnology (BIOTEC), National Biobank of Thailand, Pathum Thani, Thailand; 3 Department of Pediatrics, University of California San Diego, La Jolla, CA USA; 4 Thalassemia Research Center, Institute of Molecular Biosciences, Mahidol University, Nakhon Pathom, Thailand; 5 Skaggs School of Pharmacy and Pharmaceutical Sciences, University of California San Diego, La Jolla, CA USA; 6 Shu Chien-Gene Lay Department of Engineering, University of California San Diego, La Jolla, CA USA; 7 Department of Computer Science and Engineering, University of California San Diego, La Jolla, CA USA; 8 Halıcıoğlu Data Science Institute, University of California San Diego, La Jolla, CA USA

**Keywords:** Thalassemia, Iron overload, Microbiome, Anaemia, Microbiome

## Abstract

Recent research highlights the significant impact of the gut microbiota on health and disease. Thalassemia, a hereditary blood disorder, requires regular blood transfusions, leading to an accumulation of iron in the body. Such changes could potentially alter the intestinal microbiota, thereby increasing the susceptibility of thalassemic patients to infection. In this study, we analyzed the fecal microbiota of 70 non-transfusion-dependent (NTDT) β-thalassemia/HbE patients and 30 healthy controls. Our findings indicate that iron chelation intervention had no detectable effect on the microbiome profile of thalassemic patients. However, the cross-sectional analysis revealed that the bacterial diversity and community structure in patients were significantly less diverse and distinct compared to those of healthy subjects. Using reference frames, we were also able to demonstrate that bacterial taxa that are known to produce short chain fatty acids, from the genera *Alistipes*,* Coprococcus*, and *Oscillospira*, and those from the family *Ruminococcaceae*, were less prevalent in the patients. In contrast, bacterial taxa associated with an unhealthy gut, including the genus *Clostridium* and those from the families *Fusobacteriaceae*, *Enterobacteriaceae*, and *Peptostrptococcaceae*, were more prevalent in patients and found to be correlated with higher levels of ferritin. Collectively, these changes in the microbiota could be regarded as markers of raised ferritin levels, and therefore, awareness should be exercised as they could interfere, albeit indirectly, with the treatment of the co-morbidities of thalassemia.

**Supplementary Information:**

The online version contains supplementary material available at 10.1038/s41598-024-75456-4.

## Introduction

Thalassemia is a hereditary blood disorder caused by an excess of unpaired globin chains due to defective globin synthesis. Point mutations in the β-globin gene lead to the absence of or reduced production of β-globin, giving rise to β-thalassemia, in which the imbalance between the β- and α-globin chains results in ineffective erythropoiesis and, consequently, hemolysis^1^. Hemolytic anemia, splenomegaly, and iron overload are well-known characteristics of β-thalassemia. Severe hemolytic anemia necessitates frequent blood transfusions, a common requirement in thalassemia management^2,3^. Transfused blood contains iron, which is stored in the tissues by binding to ferritin, a protein that stores iron^4–6^. When iron content exceeds the capacity of ferritin, this free iron, together with products of free α-globin chain breakdown, catalyzes the formation of free radicles, causing a multitude of organ and cellular damage^7^. Endocrine, hepatic, and cardiac tissues are the most affected by iron deposition resulting in a serious condition associated with morbidities and, in many instances, mortality^8–10^. Therefore, iron chelation therapy plays a crucial role in contributing to the significant improvement in the survival of thalassemic patients. This therapeutic approach, typically involving iron chelators such as desferrioxamine, deferiprone, and deferasirox, is initiated after 10–20 transfusions or when serum ferritin levels exceed 1000 µg/L to prevent adverse effects of iron overload^3,9^.

Excess iron not only directly cause catastrophic effect at cellular and organs level but the iron is known to have effects on the gut microbiota composition^11–14^. An altered intestinal microbiota has been shown to be associated with chronic conditions such as obesity, irritable bowel disease, atherosclerosis, liver diseases, and liver cancer, as well as genetic disorders such as autism spectrum disorder^15–17^. In particular, iron can disrupt the balance towards the growth of infectious organisms, as shown in previous studies where iron fortification affected the balance of intestinal commensal bacteria by favoring the growth of pathogenic bacteria^18,19^. Moreover, studies in iron-overloaded thalassemia mice showed that the gut mucosal injury releases lipopolysaccharides, from Gram-negative bacteria residing in the gut, which themselves are a cause of inflammatory responses^20^ and enhanced sepsis^21^. A recent study on mice showed that both intravenous infusions and blood transfusions can cause iron overload, which raises the amount of iron in the feces and changes the gut microbiota in a way similar to dietary iron^22^. Thus, it is unsurprising that infections and sepsis are the major causes of morbidity and mortality in β-thalassemia^23,24^.

Gut inflammation and gut barrier disruption are not solely influenced by changes in the composition of gut microbiota but also by alterations in the metabolites produced by these altered microbial communities^25^. Short-chain fatty acids (SCFAs), the gut microbiota-producing metabolites, play a crucial role in preserving gut health by promoting the integrity of the intestinal barrier, therefore inhibiting the colonization of pathogenic microbes^26,27^. Additionally, SCFAs possess potent anti-inflammatory characteristics by regulating immunological responses and suppression of pro-inflammatory cytokine synthesis^28^. These roles are crucial in β-thalassemia, where excessive iron intake from recurrent blood transfusions worsens inflammation of the stomach and degeneration of the epithelium^29,30^.

These incidental studies raise an interesting question as to whether iron overloading among thalassemic patients, resulting from increased iron absorption and frequent blood transfusions, could alter the intestinal microbiome, especially those that are involved in SCFA production, and consequently explain the high rate of susceptibility to infection in these patients. In our study, we analyzed changes in the gut microbiota using fecal samples of thalassemic patients and observed significant alterations in the bacterial diversity and community structure of the gut microbiota. We also found that some bacterial taxa that are known to promote or prevent infections are significantly altered in thalassemic patients. This study also presented a correlation between iron status and unhealthy gut-associated bacteria taxa. This discovery of dysbiosis in thalassemic patients is one of the first studies on the microbiome composition in thalassemia and provides some possible insight on the prognosis and treatment outcomes of infections in thalassemic patients.

## Results

### Participant and characteristics

The study was conducted in Nakhon Pathom province, Thailand. We recruited 70 non-transfusion-dependent (NTDT) β-thalassemia/HbE patients and 30 healthy subjects. Detailed inclusion and exclusion criteria can be found in Supplementary Table 1. This study was approved by the Mahidol University ethics committee (Approved protocol number MU-CIRB 2018/181.1309 and COA No. MU-CIRB 2018/184.2610), and written informed consent was obtained from all participants. The patients were classified into three groups of iron overload based on their serum ferritin level: Low (< 1,000 ng/ml, *n* = 39), Medium (1000–2000 ng/ml, *n* = 15) and High (> 2000 ng/ml, *n* = 16). The healthy subjects were recruited from people living in the same environment as the patients (*n* = 30). The major characteristics of the cohort can be found in Table 1.

**Table 1:**
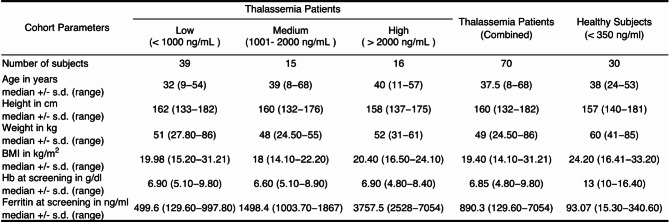
Details of the cohorts used in this study.

### Iron chelation intervention shows no detectable longitudinal effect on microbiome profile of thalassemic patients in this study

**Fig. 1:**
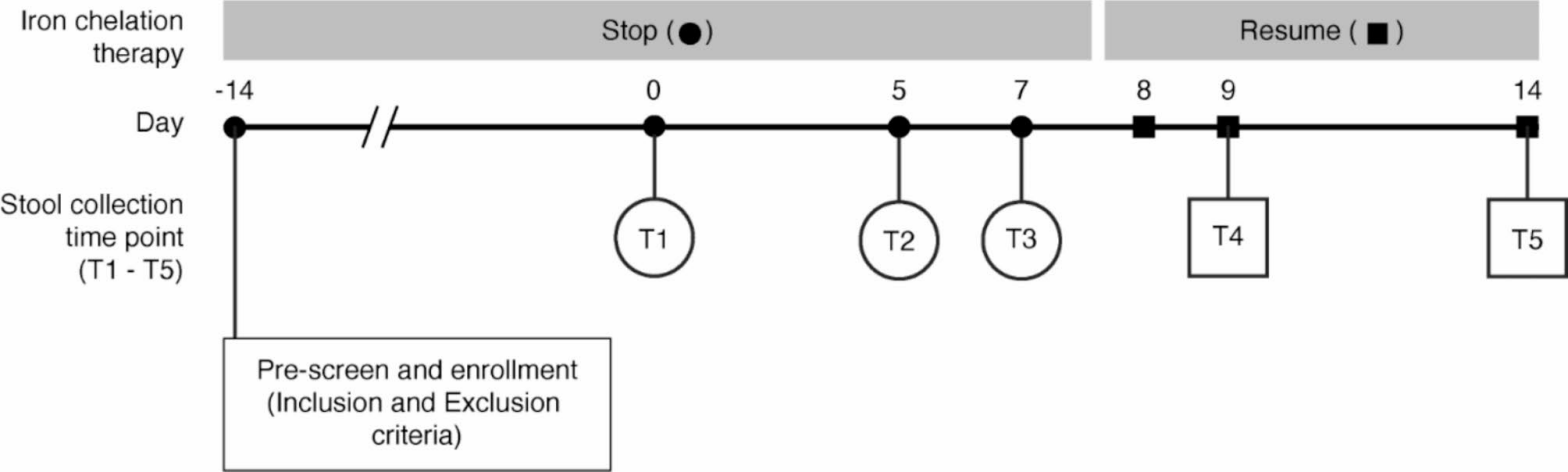
Study design. Initial screening involved a baseline health assessment, including measurements of ferritin and hemoglobin levels, conducted 14 days prior (day − 14). Following enrollment, stool samples were collected at five timepoints to assess changes in the microbiome due to iron chelation therapy. The first three samples (T1-T3) were taken on days 0, 5, and 7. After resuming iron chelation on day 7, the final two samples (T4 and T5) were collected on days 9 and 14.

One of the questions we aimed to address in this study was whether iron chelator administration has a detectable effect on a patient’s microbiome profile. Thus, we designed and conducted a longitudinal study focusing on the effect of iron chelation intervention by comparing patients’ microbiota profiles before (Stop period, T1-T3) and after (Resume period, T4-T5) iron chelation (Fig. 1). We determined the Shannon diversity index to test if the alpha diversity of patients’ microbiota between the two time periods (Stop vs. Resume) was significantly altered compared to healthy subjects. However, no significant difference in the Shannon diversity index was observed between Stop and Resume periods in all patient groups (Low, Medium, and High ferritin) when compared to the changes in the Shannon diversity index observed in the healthy subjects during the same period (Supplementary Fig. 1a, b). Also, beta diversity based on unweighted-UniFrac displayed that no significant difference was observed between Stop and Resume periods among all patient groups (Supplementary Fig. 1c, d). Altogether, these findings suggest that the effect of iron chelators on the microbiota composition of patients could not be detected in this study. Therefore, the remainder of the study will focus solely on a cross-sectional study of the microbiome profiles of thalassemia patients and healthy subjects prior to the administration of iron chelators (Stop period, T1-T3).

### The microbiome profiles of thalassemic patients are distinct and less diverse than those of healthy subjects

**Fig. 2:**
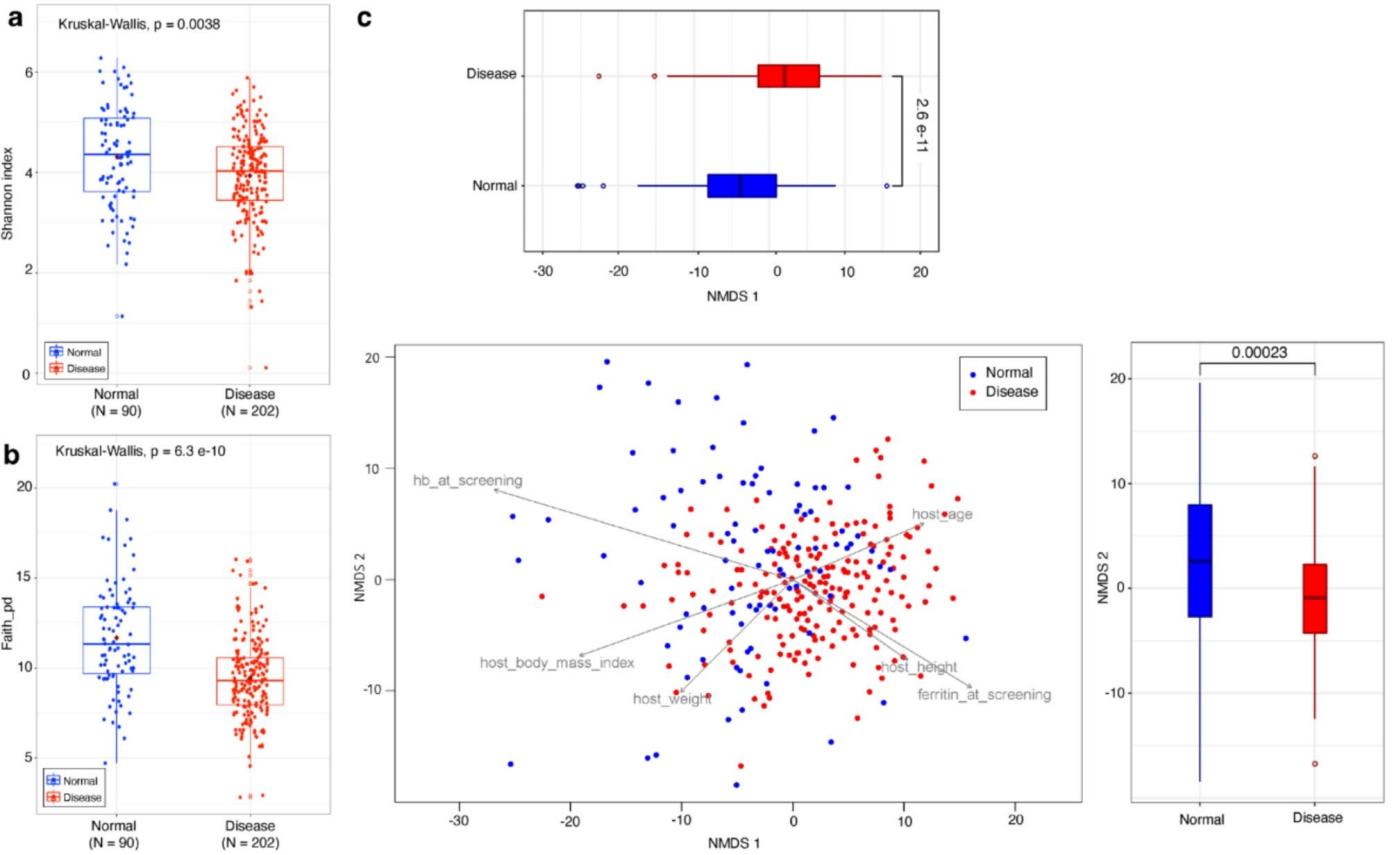
Bacterial community of thalassemia patients are less diverse and distinct compared to healthy subjects’ microbiome profile. Box plots displaying (**a**) Shannon index and (**b**) Faith’s phylogenetic diversity index in two different groups. These values were compared to others in a pairwise manner with the Kruskal-Wallis test (α = 0.05). (**c**) Non-metric multidimensional scaling (NMDS) biplot based on Aitchison distance. MetaMDS and envfit functions (vegan package) in R were calculated and used to display correlations between microbial community compositions and environment metadata. Dots represent individual samples, where blue color represents normal and red color represents disease group. Vectors (arrows) represent the direction of metadata in relation to normal or disease groups. The comparison between two groups (Normal vs. Disease) of box plots was calculated based on the Wilcoxon test.

We first investigated whether the microbiota of thalassemic patients differed from that of healthy subjects. From all 100 enrolled participants, a total of 300 samples were collected during the Stop period (T1, T2, and T3 timepoints). After data processing, the total number of qualified samples was reduced to 292 and subsequently used for the rest of the analysis in the study. The Shannon index (Fig. 2a) and Faith’s phylogenetic diversity (Fig. 2b) in healthy subjects (4.307 and 11.678, respectively) were significantly higher than those in thalassemic patients (3.937 and 9.453, respectively), suggesting that thalassemic patients have less microbial diversity than healthy subjects. Next, compositional Aitchison β diversity of samples was analyzed to elucidate the nature of community differences between thalassemic patients and healthy subjects. Non-metric multidimensional scaling (NMDS) analysis suggests that bacterial communities among two groups were significantly different (*p* = 0.001, PERMANOVA), especially along the NMDS 1 axis (Fig. 2c).

Due to the notable differences in diversity observed, we looked into what factors caused the differences in the bacterial communities between thalassemic patients and healthy subjects. While there was no significant difference in age between the patients and healthy subjects (*p* > 0.1, t-test), BMI (*p* < 0.001), ferritin level (*p* < 0.001), and hemoglobin level at screening (*p* < 0.001) were significantly different (Table 1). BMI was significantly higher in healthy subjects compared to the thalassemic patients, which is in agreement with previous studies demonstrating that the BMI of the Thai population (young men) ranges from 21.7 kg/m^2^ to 22.5 kg/m^2^^31^, while thalassemic patients normally have a lower BMI^32^. Next, correlations between microbial community composition and environmental metadata were analyzed (Fig. 2c). The results show that body mass index, weight, and HB levels at screening were negatively correlated with bacterial community composition (envfit; body mass index: *R*^2^ = 0.0923, *p*-value = 0.001, weight: *R*^2^ = 0.0455, *p*-value = 0.002, HB level: *R*^2^ = 0.1750, *p*-value = 0.001), while ferritin level, height, and age were positively correlated (envfit; ferritin level: *R*^2^ = 0.0783, *p*-value = 0.001, height: *R*^2^ = 0.0354, p-value = 0.008, and age: *R*^2^ = 0.0366, *p*-value = 0.005). Notably, the HB level at screening and the ferritin levels had a strong effect on the make-up of the microbial communities in healthy people and thalassemic patients.

## Higher level of Proteobacteria and Fusobacteria phylum observed in thalassemic patients

**Fig. 3:**
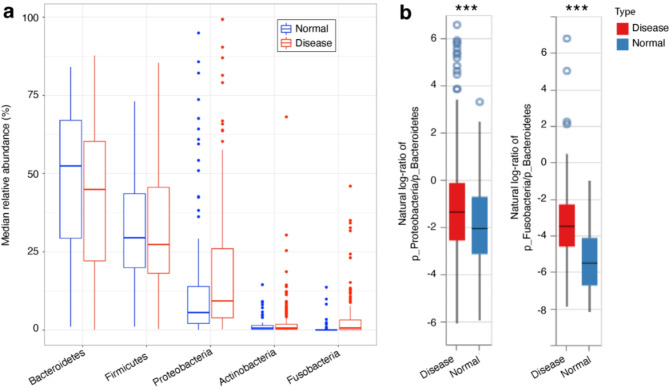
Changes in microbial population at phylum level in the normal and thalassemia groups. **(a)** A boxplot displaying the relative abundance (%) of five main phyla in normal (blue) and disease (red) groups. A Wilcoxon two-sided test was used to calculate the significance (*p*-value < 0.05). (**b**) Boxplots showing the log-ratios of phyla Proteobacteria and Fusobacteria to those of the phylum Bacteroidetes. All log–ratios are statistically significant, based on a two-sample *t*-test (*** *p* < 0.001).

Since changes at the phylum level have been used as indicators of various dysbiosis^15,33^, we next investigated changes in microbial population at the phylum level in both groups. At the phyla level (Fig. 3a), Bacteroidetes was the dominant phylum in both groups, with an average of 44.03%, followed by Firmicutes at 33.33% on average. Proteobacteria, Fusobacteria, Actinobacteria, and Verrucomicrobia were at 16.87%, 0.02%, 0.02%, and 0.01%, respectively. Significant differences in the relative abundance of phyla could be seen between the normal and diseased groups (Fig. 3a). Proteobacteria were higher in the disease group at 9%, whereas 5.59% in the normal group.

Similarly, Fusobacteria exhibited higher abundance in the diseased group at 0.65%. while it was absent (0%) in the normal group. The differences in the relative abundances of both Proteobacteria and Fusobacteria between the diseased and normal groups were statistically significant (Wilcoxon two–sided test; *P*–value < 0.05). On the other hand, differences in the relative abundance between normal and diseased groups were also observed for other phyla but were not statistically significant. This was the case for the relative abundances of Bacteroidetes, at 52.44% and 44.90%, Firmicutes at 29.49% and 28.1%, and Actinobacteria at 0.63% and 0.64%, for the normal and diseased groups, respectively. Due to the compositional effect of these sequencing datasets, Songbird and log-ratios are preferred to determine the differences between the groups^34^. Since Bacteroidetes is one of the two most abundant phyla in the human gut microbiome^35^ and also showed no significant difference between normal and thalassemic groups in our study, it was used to calculate log-ratios as a reference frame for other phyla. In agreement with the phylum relative abundance data (Fig. 3a), the log-ratios of the phyla Proteobacteria and Fusobacteria to the phylum Bacteroidetes were significantly higher in the disease group compared to the healthy group, indicating that both phyla are prevalent in thalassemic patients (Fig. 3b).

### Short-chain fatty acids (SCFA) producing bacteria are prevalent in healthy subjects, while unhealthy gut-associated bacteria are prevalent in thalassemic patients

**Fig. 4:**
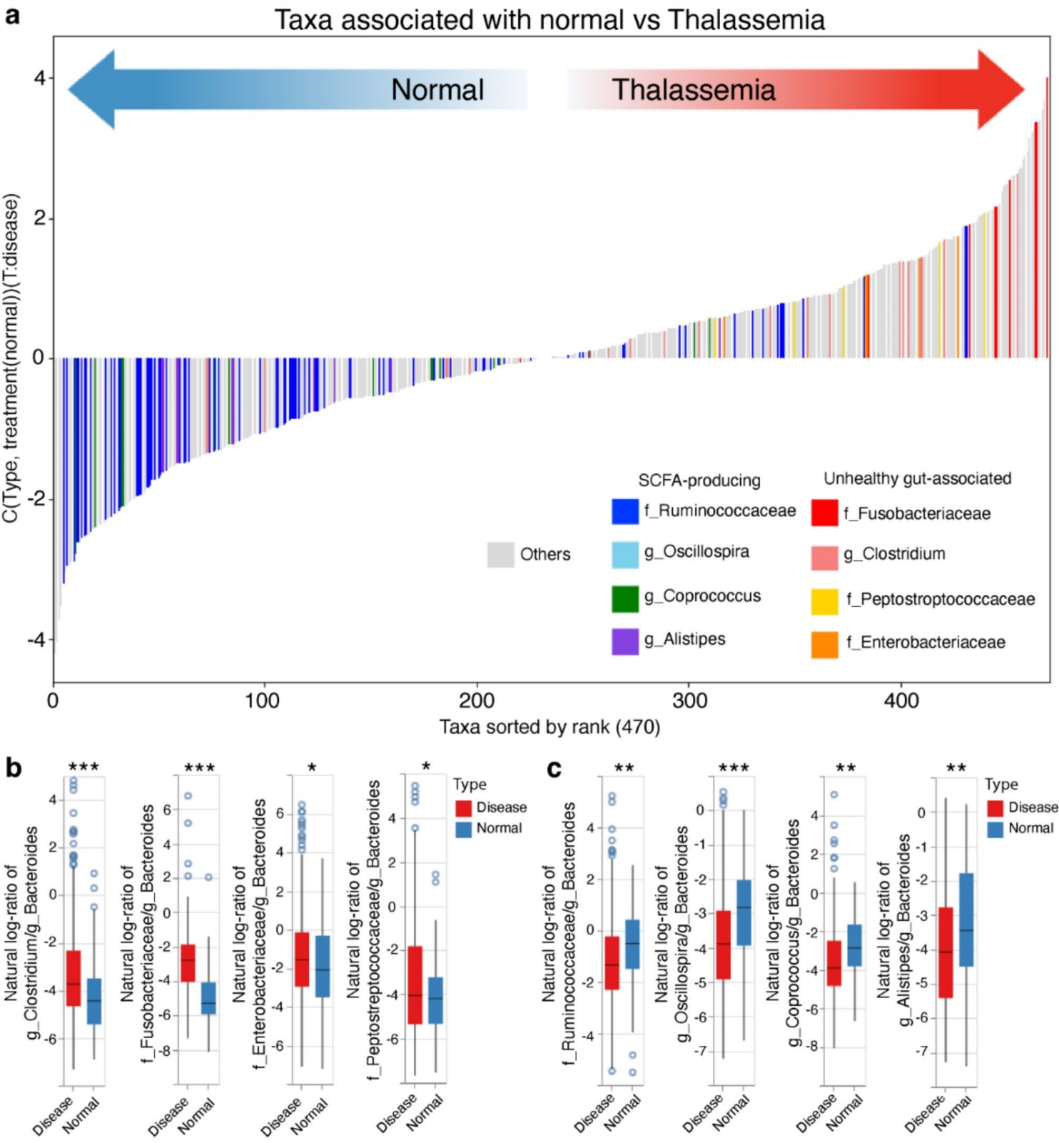
Unhealthy gut-associated, but not SCFA-producing bacteria are more prevalent in thalassemic patients. (**a**) Differential rankings of taxa associated with disease status determined by Songbird and illustrated in Qurro. Each taxon is represented by different color bars in the plot. (**b**) Boxplots showing log-ratios of different unhealthy gut-associated bacterial taxa to that of genus Bacteroides. (**c**) Boxplots showing log-ratios of different SCFA-producing bacterial taxa to those of the genus Bacteroides. All log-ratios are statistically significant, based on a two-sample *t*-test (*p < 0.05, ** *p* < 0.01 and *** *p* < 0.001).

Identifying taxonomic signatures in microbiome datasets that can infer the health status of the subject is crucial for disease marker development. In this study, we used Songbird^34^ and Qurro^36^ to find the differential ranks of each taxa, determining which taxa are prevalent in healthy subjects and which in thalassemic patients. The analysis revealed that taxa that are known to be associated with an unhealthy gut, including the genus *Clostridium*, the families *Fusobacteriaceae*, *Enterobacteriaceae*, and *Peptostrptococcaceae*, are prevalent in thalassemic patients. In contrast, taxa associated with SCFA production, such as the genus *Alistipes*^37^, *Osillospira*^38^, *Coprococcus*^39^, and the family *Ruminococcaceae*^40^, are more prevalent in the normal group (Fig. 4a). The full set of differential rank is listed in Supplementary Table S2. Since the genus *Bacteroides* was prevalent and most abundant in both groups according to the differential ranking and also showed no significant difference between the normal and disease groups (Supplementary Fig. 2), it was used as the reference frame to calculate log-ratios for other taxa. Indeed, the log-ratios of unhealthy gut-associated taxa (Fig. 4b) to the genus *Bacteroides* were significantly higher in the disease group compared to the healthy group. In contrast, log-ratios of SCFA-producing taxa were significantly lower in the disease group (Fig. 4c). Together, these findings suggest that, in healthy individuals, bacteria that produce SCFAs are common, whereas thalassemic patients tend to have a higher prevalence of unhealthy gut-associated bacteria.

## Higher ferritin levels are linked to unhealthy gut-associated bacteria abundance

**Fig. 5:**
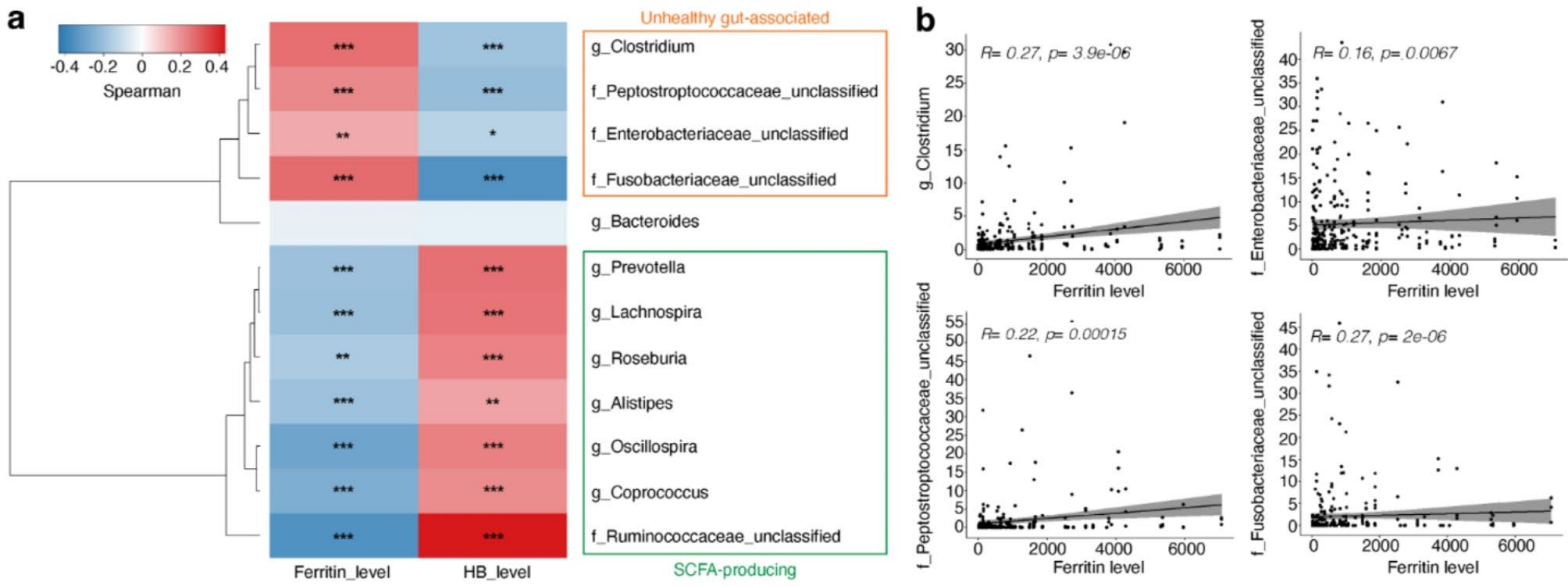
Higher ferritin level is linked to higher abundance of unhealthy gut-associated bacteria **(a)** Spearman correlations, visualized by the MicrobiomeSeq and phyloSeq packages in R, showing relative abundance of bacteria correlating with ferritin and HB (hemoglobin) levels. The adjusted p-values were calculated using Benjamini and Hochberg (***p = 0.001, **p = 0.01, *p = 0.05). **(b)** Graphs showing relative abundances (%) of unhealthy gut-associated bacteria are significantly correlated with high ferritin levels.

Given that iron-overloaded conditions are commonly observed in thalassemic patients, the increase in the unhealthy gut-associated bacteria found in patients urged us to investigate if elevated iron levels, as indicated by ferritin levels, are associated with the abundance of these bacteria. We investigated correlations between microbiota relative abundance and hematological parameters, ferritin level and HB levels, in all 292 samples, including both healthy subjects and thalassemic patients, by employing Spearman’s correlation test (Fig. 5). The results showed that ferritin level was positively correlated with the relative abundance of certain unhealthy gut-associated bacterial taxa: *Clostridium* genus, *Enterobacteriaceae* family, *Fusobacteriaceae* family, and *Peptostreptococcaceae* family (Fig. 5a and b). Conversely, bacteria known for SCFA production, i.e., *Prevotella* genus^41^, *Lachnospira* genus^42^, *Roseburia* genus^43^, *Alistipes* genus^37^, *Osillospira* genus^38^, *Coprococcus* genus^39^ and *Ruminococcaceae* family^40^, showed a negative correlation with the ferritin levels (Fig. 5a). In thalassemia, ferritin levels are known to inversely correlate with HB levels due to the pathogenicity of the disease^7^. Thus, it is unsurprising that the relationship between bacteria abundance and ferritin level is opposed to the bacteria abundance and HB levels. Together, these results suggest that ferritin levels are correlated with unhealthy gut-associated bacteria, while HB levels are correlated with SCFA-producing bacteria in participants.

## Discussion

NTDT β-thalassemia/HbE patients were specifically selected for this study due to their predominance in the patient population registered at our designated clinic. Although NTDT patients require fewer blood transfusions, they still experience a certain degree of iron overload. Furthermore, the reduced need for blood transfusions in NTDT patients is associated with a more stable clinical profile compared to those with transfusion-dependent thalassemia (TDT). This stability during the course of our investigation allowed us to minimize confounding variables related to frequent transfusions and their associated complications, thereby enabling a more accurate assessment of the microbiome alterations. Given the potential differences in the gut microbiome between NTDT and TDT patients, it is recommended that future studies focusing on TDT patients would be crucial for dissecting the specific effects of chronic iron overload and frequent transfusions on the gut microbiome in TDT patients.

In this study, we first found that the effect of iron chelators on the microbiota composition of patients could not be detected in the longitudinal study. Consistent with our findings, previous research conducted in a murine model demonstrated that treatment with deferiprone, an iron chelator, failed to alleviate gut dysbiosis in heterozygous β-thalassemia mice^44^. This can be partially attributed to the fact that iron chelators do not have direct bactericidal effects^45^. Therefore, it is not anticipated that there will be sudden alterations in the microbiome profile following the administration of an iron chelator, as would often occur after taking antibiotics^46^. Moreover, long-term treatment is recommended to achieve and maintain reduced iron levels in organs and tissues, for example, thalassemic patients receiving iron chelator therapy are required to have their liver iron concentrations monitored every 6 months and serum ferritin every 3 months, in order to observe a change in iron levels^47^. Therefore, the length of the iron chelation intervention in this study might not have been adequate to detect changes in the microbiome profile of the patients. Furthermore, the relatively low number of patients in each group (Low, Medium, and High ferritin) might undermine the analysis power of the study, resulting in the undetectable changes in diversity indexes caused by iron chelation intervention. It is well-documented that determining the minimum number of participants required to find statistically significant differences in clinical microbiome studies is a largely unresolved subject, especially when the preliminary data of the microbial diversity in the targeted population is unavailable^48^. Given the lack of prior information on the microbial diversity of the NTDT-thalassemic patients, the sample size for this study was primarily determined based on feasibility considerations. Despite this limitation, this study provides valuable baseline data on the microbial diversity of NTDT-thalassemic patients for future research investigating microbiome changes that are clinically relevant to interventions such as iron chelation therapy.

A cross-sectional study of the microbiome profile demonstrated that the gut microbiota in fecal samples from thalassemic patients exhibited lower diversity and distinctiveness compared to those from healthy subjects. Contrary to a previous study on thalassemic mouse models where iron overload did not affect the diversity of the fecal microbiome^10^, our findings align with studies on humans with various chronic conditions such as diabetes^49^, allergic asthma^50^, liver cirrhosis^51^, and inflammatory bowel disease (IBD)^52^. These studies also observed less diverse and distinct microbiota profiles among diseased groups. At the phylum level, we observed that Proteobacteria and Fusobacteria were significantly higher in thalassemic patients. Similar to our findings, the higher abundance of Proteobacteria has been found to be associated with intestinal inflammation^11^, colitis, and IBD^53^. Fusobacteria has also been found to be enriched in immunocompromised patients^54^ and patients suffering from liver cirrhosis^51^.

Regarding the changes in SCFA-producing bacteria, in this study, we observed a reduced prevalence of bacterial taxa from genera such as *Alistipes*,* Coprococcus*, and *Oscillospira*, and those from the family *Ruminococcaceae*, all of which are SCFA-producing bacteria^37–40^. The low prevalence of SCFA-producing bacteria in thalassemic patients may result in decreased SCFA production, leading to compromised intestinal immunity and diminished ability to maintain the integrity of intestinal epithelial cells^55^. Particularly, bacteria from the family *Ruminococcaceae* was reported to be significantly reduced in patients with IBD^40^, and species from the genus *Alistipes* have been shown to possess anti-inflammatory and tumor-suppressive properties, protecting the gut against colitis^37^. Both *Ruminococcaceae* and *Alistipes* were less prevalent in thalassemic patients in our study, and therefore, it is not surprising that thalassemic patients are prone to systemic infections with sepsis and liver abscess, among others, being common^23,24,56^. Additionally, as high levels of SCFAs have been shown to inhibit the growth of *Enterobacteriaceae*^57,58^, this could possibly explain the higher prevalence of *Enterobacteriaceae* in thalassemic patients. Blooms of *Enterobacteriaceae* have been known to enhance the susceptibility of the gut to pathogens such as *Clostridium difficile*^57^, which also coincides with our finding of a higher prevalence of the *Clostridium* genus thalassemic patients. On the other hand, *Lachnospiraceae*, which was known to hinder *Clostridium difficile* colonization^59^, was less prevalent in thalassemic patients in our study. Whether the bacterial taxa and their members identified in this study play significant roles in clinical outcomes, such as increased susceptibility to intestinal infections, could be an intriguing topic for future studies.

Besides, healthy subjects in many studies were shown to have a high abundance of *Lachnospira*, *Alistipes*, and *Roseburia*, suggesting their association with good health^37,42,60^. Our data showed similar results, as these genera had positive correlations with hemoglobin levels, indicating a healthy state of participants. A previous study reported that *Oscillospira* was also found to be closely related to healthy humans and that it was positively correlated with a diverse microbial environment^61^. Moreover, negative correlations have been found between *Oscillospira* and *Clostridium*, with the latter being able to produce metabolites that could render an unfavorable environment for the growth of *Oscillospira*. This report supports our findings that the relative abundance of *Clostridium* correlated with ferritin levels, indicating disease, whereas that of *Oscillospira* correlated with hemoglobin levels, indicating normal health. Additionally, we observed that *Copprococcus*, a Gram-positive coccus regarded as an indicator of high quality of life, was correlated with hemoglobin levels.

Studies in both adults and children have shown that fecal *Enterobacteriaceae* are correlated with increased levels of serum ferritin^11^. *Enterobacteriaceae* can, in turn, enhance the growth of disease-associated bacteria, particularly *Clostridium difficile*^57^. These findings are consistent with our results, as we observed a correlation between increased ferritin levels and the presence of the genus Clostridium and families *Enterobacteriaceae*. It is worth noting that, in this study, only ferritin level was used as an indicator of the systematic iron status of the participants. Thus, further studies are needed to dissect whether the prevalence of unhealthy gut-associated bacteria in gut microbiome of thalassemic patients is associated with the fecal iron level similar to the ferritin levels shown in this study.

Other limitations of the study should also be acknowledged. Firstly, it is necessary to take into account the variability in the collection and storage of fecal samples. Although previous research has demonstrated that interpersonal variations in gut microbiota profiles generally outweigh changes in microbial populations as a result of storage conditions^62^, it is widely acknowledged that the methodology used in microbiome analysis can have a significant impact on study outcomes^63–65^. Consistent protocols were implemented for all samples in this study, including DNA extraction, sequencing technologies, transit conditions, and storage temperature. However, the precise duration of sample storage prior to shipment could not be uniformly controlled. Research has shown that the composition of the microbiome can be influenced by differences in storage timing to varying degrees, depending on the study design^66,67^. As well as the variation resulting from sample storage, other participant-related factors may also contribute to the observed variations in microbiome profiles. We were unable to rigorously regulate the time of day during which patients visited the clinic and collected their samples on-site. The microbiome composition may be influenced by the timing of sample collection, as suggested by prior research^68,69^. In addition, the dietary intake of participants was not standardized, which is likely to have contributed to the individual variations in microbiome profiles. Diet is a well-established factor that influences the human microbiome, as studies have shown that dietary changes can swiftly alter the microbiome composition within days^70–72^. Consequently, although this study identified differential microbiome profiles and specific dominant species that are associated with iron overload in thalassemic patients, it is crucial to take into account the potential covert effects of sample handling and storage as well as the absence of control over the food consumption of participants during the study period when interpreting the microbiome shifts that were observed in this study.

The potential clinical applications of microbiome profiling in the prevention and treatment of diseases are becoming more evident, paving a way for personalized therapeutic interventions^73,74^. For example, the involvement of the gut-liver axis in liver injury caused by excess iron was highlighted, indicating that targeting specific gut microbes could alleviate liver damage^73^. Furthermore, clinical applications could be extended to monitoring of microbiome profiles as indicators of disease progress, particularly in the management of sepsis, where the gut microbiome has been recognized as a promising therapeutic target^74,75^. In particular, profiling the microbiota in β-thalassemia patients, where excessive iron intake compromises gut integrity, could contribute to the identification of individuals with an increased susceptibility to severe mucositis and sepsis^20,21^. Our results indicate that thalassemic patients have a higher prevalence of pathogenic bacteria, which are also associated with elevated ferritin levels. Therefore, it is crucial to regard these bacteria as indicators of increased ferritin levels. When the microbiome of thalassemic patients exhibits an abundance of these bacteria, caution is warranted, as they may indirectly impact the outcomes of treating thalassemia co-morbidities. In order to restore healthy gut microbiota and prevent pathogen translocation that leads to systemic infections, future research should prioritize the development of microbiome-based diagnostics and therapeutics, such as phage therapy, probiotics, or prebiotics^76–78^.

## Materials and methods

### Study design and sample collection

For the primary screening process, all participants were reviewed at baseline (-14 day, Fig. 1) for health assessment, measuring of ferritin and hemoglobin levels according to a previous study^79^. Once enrolled, all thalassemic patients were asked to discontinue iron chelation for 14 days before the first sample collection. Stool samples were collected at five different timepoints to evaluate longitudinal microbiome alteration caused by iron chelation therapy intervention. Stool samples from the first three time points (T1-T3) were collected on days 0, 5, and 7, respectively. After day 7, iron chelation therapy was resumed for patients. Stool samples for the last two time points, T4 and T5, were collected at days 9 and 14, respectively. For healthy subjects, all five time points were collected within the same time interval as those of the patients, but without any intervention. The experimental design is summarized in Fig. 1. Stool collection procedures were carried out using BD BBL™ CultureSwab™ EZ II. Once collected, samples were kept at 4 °C during transport and frozen at − 20 °C within 1 h. Since recruitment spanned over the course of 12 months, some samples were kept frozen longer than the others. However, this was consistent across all groups for comparison purposes.

### DNA extraction

Procedure for the extraction of genomic DNA from fecal samples were modified from MagAttract^®^ PowerSoilⓇ DNA KF Kit (384) (QIAGENⓇ catalogue: 27000-4-KF). The sample swabs were processed in each well of a PowerBead DNA plate followed by the robotic program on the KingFisher Flex Purification System^80^.

### 16 S sequencing

An amplicon library was constructed, using the previously extracted genomic DNA, by amplifying the V4 region of 16S ribosomal RNA gene. The primers used for this amplification were FWD 515F 5’ – GTGYCAGCMGCCGCGGTAA – 3’ and REV 806R 5’ – GGACTACNVGGGTWTCTAAT – 3’ (https://earthmicrobiome.org/protocols-and-standards/16s/). PCR reaction was carried out on a thermocycler, and the amplicon obtained was quantified and sequenced on Illumina Platform, in accordance with the previous protocol (10.17504/protocols.io.nuudeww**)** and the previous study^81^.

### HB level and ferritin level assessment

Three mL of an EDTA blood tube were used for hematological and Hb analyses using the automatic HPLC (Bio Rad, Variant II system), while 4 mL were used for serum ferritin measurement using immunoenzymatic kits (DiaMetra, Segrate, Italy).

### Bioinformatic analysis

Raw sequencing data in FASTQ format were processed using QIIME 2 (version 2019.10)^82^ based on Deblur^83^, which provides single-nucleotide resolution based on error profiles within samples. First, paired-end FASTQ reads from each sample were merged using vsearch^84^ to join paired-end reads. Next, merged reads were filtered based on read quality using QIIME 2’s built-in quality-filter q-score-joined script. Then, filtered reads were processed with Deblur, using a trim length of 250 base pairs to obtain amplicon sequence variants (ASV). A phylogenetic tree was then generated for phylogenetic diversity analyses. A phylogenetic tree was built using the q2-fragment-insertion plugin^85^ based on Greengenes 99% identity database. The amplicon sequence variants (ASVs) were subsampled, based on the lowest count sequences (rarefied to 2000 to minimize the effects of sequencing depth) for alpha and beta analysis using the core-metrics-phylogenetic method.

Alpha diversity metrics were used to estimate microbial diversity based on Shannon and Faith’s phylogenetic diversity between two groups. Pairwise comparisons of alpha diversity were calculated using a Kruskal-Wallis test. Moreover, the Shannon diversity index was also estimated to test whether alpha diversity changed significantly between two different time periods (stop and resume) according to ferritin level with q2-longitudinal plugin^86^.

Beta diversity analysis, including robust Aitchison and unweighted-UniFrac distance, was calculated using the beta-group-significance command in QIIME 2. Permutational multivariate analysis of variance (PERMANOVA) based on unweighted-UniFrac was also used to analyze statistical differences in beta diversity with QIIME 2. Moreover, a non-metric multidimensional scaling (NMDS) plot, displayed as a biplot, showed correlations between microbial community composition and environment metadata. NMDS based on Aitchison distance with biplot, was calculated by metaMDS and envfit functions (vegan package) in R^87^. A pairwise comparison of beta diversity based on Aitchison distance was calculated using the Wilcoxon test. For taxonomy analysis, it was assigned to ASVs using Greengenes (version 13_8) database classifier. A non-parametric two-group comparison was performed with the Wilcoxon test.

The differential rankings of taxa associated with thalassemia and the normal group were determined using Songbird^34^. ASVs generated by QIIME 2 were used as input for Songbird to rank taxa associations with the different groups, with normal samples serving as a reference. Qurro^36^ was also used to calculate and visualize the differential ranks of taxa associated with thalassemia versus the normal group. Moreover, correlations between the relative abundance of genus and ferritin level were calculated based on Spearman’s correlation, and *p*-value were adjusted using the Benjamini and Hochberg method^88^. The correlation coefficients were visualized using MicrobiomeSeq (https://github.com/umerijaz/microbiomeSeq.git) and the phyloSeq package in R^89^.

## Electronic supplementary material

Below is the link to the electronic supplementary material.

Supplementary Material 1

## Data Availability

The data presented in this study are available in the manuscript and supplementary materials. Other data are available on request from the corresponding author. Some data is not publicly available due to the Personal Data Protection Regulation.
