# Supplementary material for: Differential gut microbiota composition in β-Thalassemia patients and its correlation with iron overload
Source: Sci Rep. 2024 Oct 11;14:23858. doi: 10.1038/s41598-024-75456-4 (PMC11470119; doi:10.1038/s41598-024-75456-4)
Supplement: Supplementary file 1 — Supplementary Material 1 [file 41598_2024_75456_MOESM1_ESM.pdf]

Supplemental materials for

Title

Differential Gut Microbiota Composition in β-Thalassemia Patients and Its Correlation with Iron Overload

Authors

Poochit Nonejuie[1], Alisa Wilantho[2], Daniel McDonald[3], Htut Htut Htoo[1], Jenjira Chalerm[4], Anupriya Tripathi[3,5], Chumpol Ngamphiw[2], Sissades Thongsima[2], Rob Knight[3,6,7,8], Kittiphong Paiboonsukwong[4], Suthat Fuchareon[4,*]

[1] Institute of Molecular Biosciences, Mahidol University, Nakhon Pathom, Thailand
[2] National Biobank of Thailand, National Center for Genetic Engineering and Biotechnology (BIOTEC), National Science and Technology Development Agency (NSTDA), Pathum Thani, Thailand
[3] Department of Pediatrics, University of California San Diego, La Jolla, CA, USA.
[4]Thalassemia Research Center, Institute of Molecular Biosciences, Mahidol University, Nakhon Pathom, Thailand
[5] Skaggs School of Pharmacy and Pharmaceutical Sciences, University of California San Diego, La Jolla, CA, USA
[6] Center for Microbiome Innovation, Jacobs School of Engineering, University of California San Diego, La Jolla, CA, USA
[7]Department of Computer Science and Engineering, Jacobs School of Engineering, University of California San Diego, La Jolla, CA, USA.
[8]Department of Bioengineering, University of California San Diego, La Jolla, CA, USA.

* Correspondence: suthat.fuc@gmail.com

This PDF file includes:

Supplementary figure 1 and 2
Supplementary table 1and 2

a

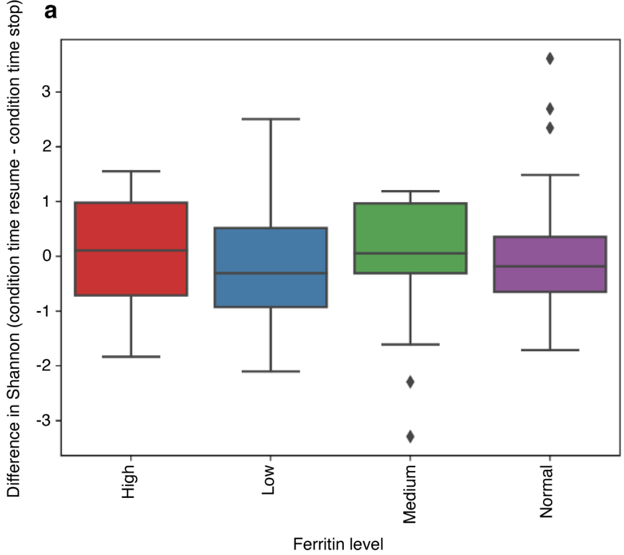

b

Group A	Group B	Mann-Whitney U	
		P-value	FDR P-value
High	Normal	0.5110	0.7665
Low	Normal	0.9085	0.92128
Medium	Normal	0.4926	0.766479

c

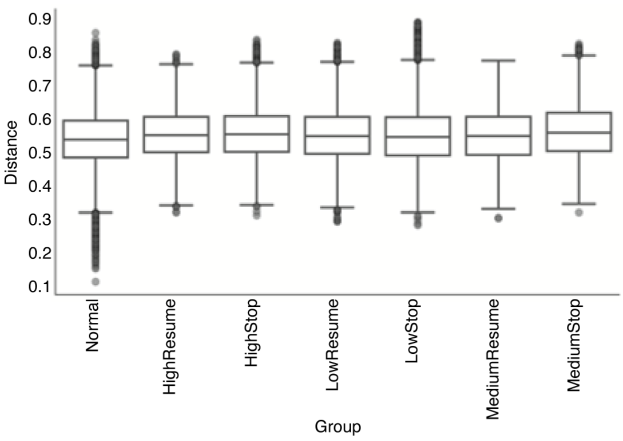

d

Group A	Group B	pseudo-F	p-value	q-value
Medium Resume	Medium Stop	0.483352	0.973	1
Low Resume	Low Stop	0.228183	1	1
High Resume	High Stop	0.455414	0.989	1

Supplementary figure 1.

Iron chelation intervention show no significant longitudinal effect on alpha and beta diversity index of thalassemia patients' microbiota in this study

a) Boxplot showing the difference of Shannon diversity index between two time periods (Stop vs Resume) in different groups; Thalassemia patients with high ferritin (High), medium ferritin (Medium), low ferritin (Low) and healthy subjects (Normal). b) Pairwise comparison of the difference of Shannon diversity between the two time periods (Stop vs Resume) in subjects with different levels of ferritin and healthy subjects. Pairwise comparison was done using Mann-Whitney U test. c) Box plot displayed unweight-UniFrac between 'stop' and 'resume' in each group with beta-group-significance function

d) The pairwise permanova results (permutations = 999) of unweighted UniFrac beta diversity across ferritin level between stop and resume periods.

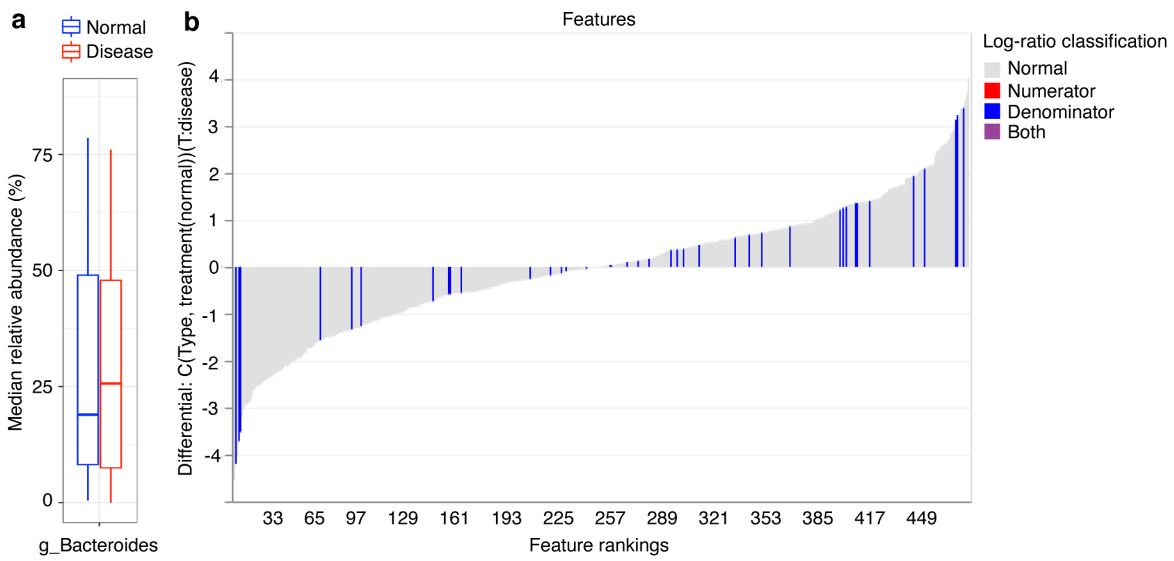

Supplementary figure 2.

Bacteroides are prevalent in both groups and showed no significant difference between normal and thalassemia groups.

a) Boxplot shows median relative abundance (%) of Bacteroides in normal and disease conditions. b) Differential ranking plot produced in Qurro, showing the prevalence of genus Bacteroides in healthy (normal) and disease conditions, determined by Songbird.

Supplementary table 1 Inclusion and exclusion criteria of the participants in this study

Inclusion criteria	Exclusion criteria
1.Diagnosis of β-thalassemia/Hb E NTDT as confirmed by hemoglobin analysis<br>2.Average total hemoglobin levels between 6.0 and 9.0 g/dL<br>3.Age 7- 55 years<br>4.Female patients of reproductive potential must have a negative serum pregnancy (b-HCG) test at screening and a negative urine pregnancy test prior to initiation of treatment<br>5.Patients who are transfusion-requiring but not dependent may be offered the opportunity to enroll	1.In the medical opinion of the Principal Investigator (e.g., due to medical history or findings upon examination), the patient is not an appropriate candidate for the study<br>2.Patient on a chronic transfusion program defined as regular transfusions every 2-8 weeks<br>3.Patient has acute or chronic infection<br>4.Patient has any evidence/suspicion of cancer of any type<br>5.Patient has flank diabetes mellitus under treatment with hypoglycemic agents<br>6.Pregnant woman<br>7.Evidence of alcohol abuse or the use of illegal drugs;<br>8.Screening laboratory result indicating HIV-positivity, or previously diagnosed with AIDS, AIDS related complex, or other immunodeficiency<br>9.Laboratory result indicating hepatitis C and/or hepatitis B infection<br>10.Unapproved concomitant drug therapy including iron supplementation and antibiotics anytime within the last 2 months prior to baseline sample collection to the time of completion of the study period

Supplementary table 2 Full list of 470 sorted differential ranking of bacterial taxa in thalassemic patients and healthy subjects

Feature ID	intercept	C(type, treatment(normal))(T:disease)	Taxon
8e90ac2f0df6304022cff8cd278f8218	1.858495653	-4.541576693	k__Bacteria; p__Firmicutes; c__Clostridia; o__Clostridiales; f__Lachnospiraceae; g__Lachnospira; s__
8ad4a56a5f526df40d97b371ba0ef33e	1.001441062	-4.201838801	k__Bacteria; p__Bacteroidetes; c__Bacteroidia; o__Bacteroidales; f__Bacteroidaceae; g__Bacteroides; s__eggerthii
5767b6d35eaaeafb23a1d7c76f6fdd35	0.129324973	-4.042519877	k__Bacteria; p__Firmicutes; c__Clostridia; o__Clostridiales; f__Lachnospiraceae
81d0ccbcad8debabdb9d3a1be8b64aae	2.696311534	-3.715864966	k__Bacteria; p__Bacteroidetes; c__Bacteroidia; o__Bacteroidales; f__Bacteroidaceae; g__Bacteroides; s__
0dca59bf326289459f112817023b0a1b	0.761735261	-3.521972964	k__Bacteria; p__Bacteroidetes; c__Bacteroidia; o__Bacteroidales; f__Bacteroidaceae; g__Bacteroides
79bdd1b666f32bea849295d02c3c1494	0.130263627	-3.201008151	k__Bacteria; p__Firmicutes; c__Clostridia; o__Clostridiales; f__Ruminococcaceae; g__Ruminococcus; s__
317d12e40d388da410a8cca6e703c423	1.512372792	-3.044234584	k__Bacteria; p__Bacteroidetes; c__Bacteroidia; o__Bacteroidales; f__Prevotellaceae; g__Prevotella; s__
defcd4620b2368ac1916885bbfb51c9e	0.422970355	-2.94568331	k__Bacteria; p__Firmicutes; c__Clostridia; o__Clostridiales; f__Ruminococcaceae
d94a05561f7643eeb4a75f59435df2df	-0.809775293	-2.933707068	k__Bacteria; p__Actinobacteria; c__Coriobacteriia; o__Coriobacteriales; f__Coriobacteriaceae
35b935487f7ac842989a43f2ec2ac517	2.075269878	-2.892649481	k__Bacteria; p__Bacteroidetes; c__Bacteroidia; o__Bacteroidales; f__[Barnesiellaceae]
493f8eda6b0c6018dd29ea027472e254	-0.913958013	-2.885371039	k__Bacteria; p__Firmicutes; c__Clostridia; o__Clostridiales; f__Ruminococcaceae; g__; s__
64032e2f2fa5269bd7d15eb19bdc94a9	1.27341038	-2.794664691	k__Bacteria; p__Firmicutes; c__Clostridia; o__Clostridiales; f__Lachnospiraceae; g__Coprococcus; s__eutactus
f2c958dfcac880c2f53a47e114d994d8	1.835126102	-2.625789235	k__Bacteria; p__Firmicutes; c__Clostridia; o__Clostridiales; f__Ruminococcaceae; g__Oscillospira; s__
5610b04f19aaac78ac2469a3feb707e9	1.102927029	-2.615875552	k__Bacteria; p__Firmicutes; c__Clostridia; o__Clostridiales; f__Lachnospiraceae; g__Lachnospira; s__
0b9e4131f2e44d54f122342f66114d65	-0.588514268	-2.554584572	k__Bacteria; p__Firmicutes; c__Clostridia; o__Clostridiales; f__Ruminococcaceae
e0a25143b7f2e59d7266ee802d1aeb61	3.22611183	-2.540949414	k__Bacteria; p__Firmicutes; c__Clostridia; o__Clostridiales; f__Ruminococcaceae; g__Oscillospira; s__
7859f0d526ad56fb4d8383d136b3bae6	-0.051141679	-2.516070912	k__Bacteria; p__Firmicutes; c__Clostridia; o__Clostridiales; f__Ruminococcaceae; g__Faecalibacterium; s__prausnitzii
6d59e45524751443fb12c13822f06712	0.077080548	-2.509744237	k__Bacteria; p__Firmicutes; c__Clostridia; o__Clostridiales; f__Clostridiaceae
63027647b049a274c881df23a4611b35	-1.075922429	-2.463429282	k__Bacteria; p__Firmicutes; c__Clostridia; o__Clostridiales; f__Ruminococcaceae
cc4ff6e54743c750e2efa569086a38cf	-1.424849927	-2.435456822	k__Bacteria; p__Firmicutes; c__Clostridia; o__Clostridiales; f__; g__; s__
00979c6b417b7978c1c656e263cdd054	1.030101121	-2.414161751	k__Bacteria; p__Firmicutes; c__Clostridia; o__Clostridiales; f__Lachnospiraceae; g__Coprococcus; s__
33b68099f712688fce3a89de16ad7fc0	-0.444211423	-2.413829634	k__Bacteria; p__Bacteroidetes; c__Bacteroidia; o__Bacteroidales; f__Rikenellaceae; g__; s__
8a8122435aec8f22921d2dc0f67a8adc	0.765488446	-2.362121651	k__Bacteria; p__Bacteroidetes; c__Bacteroidia; o__Bacteroidales; f__Rikenellaceae; g__; s__
5658ec7be5170db093c841fc74e8635e	2.771953404	-2.351635525	k__Bacteria; p__Bacteroidetes; c__Bacteroidia; o__Bacteroidales; f__S24-7; g__; s__
4260927e3b266486b5d210135f0ef4f6	-0.528375327	-2.34682853	k__Bacteria; p__Firmicutes; c__Clostridia; o__Clostridiales; f__[Mogibacteriaceae]; g__; s__
eca8c0c9eb78c42c73852968a67ae7f1	0.990554869	-2.304180691	k__Bacteria; p__Firmicutes; c__Clostridia; o__Clostridiales; f__Ruminococcaceae
bb221f6dc3c048ed61763de34f2d78a3	0.285196841	-2.271052907	k__Bacteria; p__Firmicutes; c__Clostridia; o__Clostridiales; f__; g__; s__
715414e8fb48ae19fc52e4fccf9ad97d	3.562526107	-2.24946029	k__Bacteria; p__Bacteroidetes; c__Bacteroidia; o__Bacteroidales; f__Prevotellaceae; g__Prevotella; s__stercorea

d4b257db9faad8674d68540e2c755d40	-0.303759277	-2.247502635	k__Bacteria; p__Firmicutes; c__Clostridia; o__Clostridiales; f__Ruminococcaceae
e803ff46adaa0fa149ef151b082378a0	-0.788417756	-2.208027432	k__Bacteria; p__Firmicutes; c__Clostridia; o__Clostridiales; f__Ruminococcaceae; g__; s__
db8e959b880d856d2d4da134a444851a	-0.192862928	-2.205124924	k__Bacteria; p__Firmicutes; c__Clostridia; o__Clostridiales; f__Lachnospiraceae
87368903a585e254843c93dbbe0b59de	2.519095421	-2.16834719	k__Bacteria; p__Firmicutes; c__Clostridia; o__Clostridiales; f__Ruminococcaceae; g__; s__
cd74125ad53d33e6980231aa218ccc5c	0.993696988	-2.124041388	k__Bacteria; p__Firmicutes; c__Clostridia; o__Clostridiales; f__Ruminococcaceae
c3c8d537f5afdd76af1dd96e7e974368	2.697948814	-2.117926667	k__Bacteria; p__Firmicutes; c__Clostridia; o__Clostridiales; f__Lachnospiraceae; g__Coprococcus; s__
de4e439ce9eac4a725a359552679d633	0.66757828	-2.112953971	k__Bacteria; p__Bacteroidetes; c__Bacteroidia; o__Bacteroidales; f__Porphyromonadaceae; g__Parabacteroides; s__gordonii
67bf09f04c0c07814918c6f90c97f123	0.622443974	-2.090469668	k__Bacteria; p__Tenericutes; c__Mollicutes; o__RF39; f__; g__; s__
03d8e6ee72e116c25595ba1d528e4e83	-1.21778053	-2.039345811	k__Bacteria; p__Firmicutes; c__Clostridia; o__Clostridiales; f__[Mogibacteriaceae]; g__; s__
76d4dadc006ff1d530d38dd3cfac300c	1.746416867	-2.028316329	k__Bacteria; p__Bacteroidetes; c__Bacteroidia; o__Bacteroidales; f__Rikenellaceae; g__; s__
60f0a102e1b3118286adc774264b5b7d	-0.850654542	-1.992282937	k__Bacteria; p__Firmicutes; c__Bacilli; o__Lactobacillales; f__Leuconostocaceae; g__Leuconostoc; s__
9104115edfeae473b2c9402cfb9a7ea1	-1.629860818	-1.98478801	k__Bacteria; p__Firmicutes; c__Clostridia; o__Clostridiales; f__Lachnospiraceae
cca4cb9f50234e4f03c31239c5fdd204	-0.445159375	-1.955201457	k__Bacteria; p__Firmicutes; c__Clostridia; o__Clostridiales; f__Ruminococcaceae; g__Oscillospira; s__
a5eb202d91e02ef2a85055c2a7e1085b	-0.355659902	-1.941776107	k__Bacteria; p__Firmicutes; c__Clostridia; o__Clostridiales; f__Ruminococcaceae; g__Oscillospira; s__
26f6853f46b06854fe5418317a261fb8	-0.14750117	-1.935264895	k__Bacteria; p__Firmicutes; c__Clostridia; o__Clostridiales; f__Ruminococcaceae
bc8fb99545fba27899a16f1ade967276	0.327402652	-1.917037795	k__Bacteria; p__Firmicutes; c__Clostridia; o__Clostridiales; f__Lachnospiraceae
c2426c44f4a41fb746a537c95a60c7bc	0.07387501	-1.873659918	k__Bacteria; p__Bacteroidetes; c__Bacteroidia; o__Bacteroidales; f__S24-7; g__; s__
262ba8fff46ca0c6fee6af217feea7df	1.286897719	-1.827965567	k__Bacteria; p__Firmicutes; c__Clostridia; o__Clostridiales; f__Ruminococcaceae
bd3c45d86ab338880235c8db92437c9e	-0.019088447	-1.815542767	k__Bacteria; p__Firmicutes; c__Clostridia; o__Clostridiales; f__Ruminococcaceae; g__; s__
04a802674683d317a8c7484ce2407ce0	1.318048537	-1.736174176	k__Bacteria; p__Firmicutes; c__Clostridia; o__Clostridiales; f__Ruminococcaceae; g__Oscillospira; s__
0d6bce2909152ecd402d6905c94ac4e9	0.948081792	-1.733069728	k__Bacteria; p__Firmicutes; c__Clostridia; o__Clostridiales; f__Ruminococcaceae; g__Oscillospira; s__
470dd02996e31255357eeeaa3672b469	2.288779795	-1.725180934	k__Bacteria; p__Firmicutes; c__Clostridia; o__Clostridiales; f__Lachnospiraceae
3cc6cc9dfd4acb48c522fd2e6b28c2fb	1.183602155	-1.695977996	k__Bacteria; p__Firmicutes; c__Clostridia; o__Clostridiales; f__Ruminococcaceae
469a17791a5c4653e8fa48723b670ebc	-1.507741391	-1.670487473	k__Bacteria; p__Firmicutes; c__Clostridia; o__Clostridiales; f__Ruminococcaceae; g__Oscillospira; s__
6d553cca181e178d2d235497f2ae2834	-0.530457198	-1.622941086	k__Bacteria; p__Bacteroidetes; c__Bacteroidia; o__Bacteroidales; f__Rikenellaceae; g__Alistipes; s__massiliensis
260f7cee8a8cf443bb7491dcf161241e	-1.354246556	-1.595614503	k__Bacteria; p__Bacteroidetes; c__Bacteroidia; o__Bacteroidales; f__[Odoribacteraceae]; g__Butyricimonas; s__
dd7975c60b489aecfc7f77523ba588bc	-0.899307668	-1.593080352	k__Bacteria; p__Firmicutes; c__Clostridia; o__Clostridiales; f__Ruminococcaceae; g__Ruminococcus; s__
30dd9fd45122f82c12b166a042d1eaf9	2.139342726	-1.564950535	k__Bacteria; p__Bacteroidetes; c__Bacteroidia; o__Bacteroidales; f__Bacteroidaceae; g__Bacteroides; s__eggerthii
e73d7f3f0353d7c592d2e13108551f31	1.351702512	-1.546026538	k__Bacteria; p__Bacteroidetes; c__Bacteroidia; o__Bacteroidales; f__[Barnesiellaceae]

2a89363f0b2ca69bb6cc0fb4c53c3971	-1.569509446	-1.53225095	k__Bacteria; p__Firmicutes; c__Clostridia; o__Clostridiales; f__Lachnospiraceae; g__Blautia
f357434f62c6834e060ea74342033ff9	0.328646243	-1.518739293	k__Bacteria; p__Firmicutes; c__Clostridia; o__Clostridiales; f__Lachnospiraceae; g__Anaerostipes; s__
6789c0a42f9d3fafb03fb3c2ffba7df8	0.923699677	-1.502229521	k__Bacteria; p__Bacteroidetes; c__Bacteroidia; o__Bacteroidales; f__Rikenellaceae; g__Alistipes; s__indistinctus
6013b9e15b35c4e724eb0c323c05f9d3	1.287744105	-1.495411465	k__Bacteria; p__Firmicutes; c__Clostridia; o__Clostridiales; f__Ruminococcaceae
0bb0e1802451aace7137a4d598f775f5	0.197394908	-1.491176675	k__Bacteria; p__Bacteroidetes; c__Bacteroidia; o__Bacteroidales; f__[Odoribacteraceae]; g__Butyricimonas; s__
299c04ddea7a3e8ebe6032dcfedb0369	-1.405594289	-1.487347434	k__Bacteria; p__Firmicutes; c__Clostridia; o__Clostridiales; f__Ruminococcaceae; g__Ruminococcus; s__
c7932b17e87572a0ef8eda8620e24407	0.576561272	-1.482544491	k__Bacteria; p__Firmicutes; c__Clostridia; o__Clostridiales; f__Ruminococcaceae; g__Ruminococcus
ee94b042fce1e0d75af093d7d1b1d340	2.15756315	-1.46731765	k__Bacteria; p__Firmicutes; c__Clostridia; o__Clostridiales; f__Ruminococcaceae; g__Ruminococcus
3cfa76e0da34a56a641ca33770efb588	-0.049058616	-1.450105498	k__Bacteria; p__Firmicutes; c__Clostridia; o__Clostridiales; f__Lachnospiraceae; g__Lachnospira; s__
664e750d411cfeb18b61f044356d626d	-1.375431955	-1.422832558	k__Bacteria; p__Firmicutes; c__Clostridia; o__Clostridiales; f__[Tissierellaceae]; g__1-68; s__
512a7900c480a80d574bd6903a2a3d11	-1.160413682	-1.406601498	k__Bacteria; p__Proteobacteria; c__Betaproteobacteria; o__Burkholderiales; f__Oxalobacteraceae; g__Oxalobacter
8a64e8e18be5e17d8ab9d76bb40f65d4	0.416545451	-1.401349852	k__Bacteria; p__Firmicutes; c__Clostridia; o__Clostridiales; f__Lachnospiraceae
f1aba425dcbf37c22bf4f9849be2c790	2.063519776	-1.401080916	k__Bacteria; p__Bacteroidetes; c__Bacteroidia; o__Bacteroidales; f__Prevotellaceae; g__Prevotella; s__copri
981d9dbb4041e6e95ae1202308fda899	0.217421115	-1.382914851	k__Bacteria; p__Proteobacteria; c__Betaproteobacteria; o__Burkholderiales; f__Alcaligenaceae; g__Sutterella; s__
bc53814d35b2f862562a1e865e923b33	-0.739017427	-1.369660924	k__Bacteria; p__Firmicutes; c__Erysipelotrichi; o__Erysipelotrichales; f__Erysipelotrichaceae; g__Holdemania; s__
34cc6c83a992b50d83fbb3b3cf7719a9	0.611480773	-1.369352648	k__Bacteria; p__Bacteroidetes; c__Bacteroidia; o__Bacteroidales; f__[Barnesiellaceae]; g__; s__
73ef8be8ee0616f92bca9b5f4f9f4438	-0.372662961	-1.355011056	k__Bacteria; p__Firmicutes; c__Clostridia; o__Clostridiales; f__Clostridiaceae; g__Clostridium; s__paraputrificum
0be8f550cd7548af59032ee27688a2c7	0.318501294	-1.354664157	k__Bacteria; p__Firmicutes; c__Clostridia; o__Clostridiales; f__Ruminococcaceae; g__Ruminococcus; s__callidus
b4d6edf2fed7f3c5477d370559a3a9cf	1.166183293	-1.336910556	k__Bacteria; p__Bacteroidetes; c__Bacteroidia; o__Bacteroidales; f__Bacteroidaceae; g__Bacteroides
e13823e500387439450b3826ea191948	-1.422235429	-1.327925275	k__Bacteria; p__Firmicutes; c__Clostridia; o__Clostridiales; f__Ruminococcaceae; g__Oscillospira; s__
5d1a0dbea138ebdb64ae27f1cfe749f4	-0.954352796	-1.313343117	k__Bacteria; p__Firmicutes; c__Clostridia; o__Clostridiales; f__Lachnospiraceae; g__Coprococcus; s__
305b6e6f59c05104e9f7d999ec96ae90	-2.103903711	-1.312011311	k__Bacteria; p__Firmicutes; c__Clostridia; o__Clostridiales; f__Ruminococcaceae; g__Ruminococcus; s__
0f885196460e86cbfd55b2c625b77020	1.036632121	-1.300911734	k__Bacteria; p__Firmicutes; c__Clostridia; o__Clostridiales; f__Veillonellaceae; g__Phascolarctobacterium; s__
a231814083060142816974207844eff2	1.463573158	-1.29785497	k__Bacteria; p__Bacteroidetes; c__Bacteroidia; o__Bacteroidales; f__Rikenellaceae
6797371940252066205fbd1b85d623ce	0.602781594	-1.265145133	k__Bacteria; p__Bacteroidetes; c__Bacteroidia; o__Bacteroidales; f__Bacteroidaceae; g__Bacteroides; s__eggerthii
1dc7907fcf2ce8955a6f6ac56b96fca0	0.544986308	-1.236901353	k__Bacteria; p__Bacteroidetes; c__Bacteroidia; o__Bacteroidales; f__Prevotellaceae; g__Prevotella; s__copri
0e1dcba2a26611bacba2adfc23ac6418	-0.402242124	-1.228350947	k__Bacteria; p__Firmicutes; c__Clostridia; o__Clostridiales; f__Lachnospiraceae; g__Coprococcus; s__eutactus
dbd3c8db1141dcb28ac371f199159b78	-0.214241922	-1.225285361	k__Bacteria; p__Proteobacteria; c__Alphaproteobacteria; o__RF32; f__; g__; s__

961ce3638f9fdd87503a442460475401	-0.515679061	-1.21539099	k__Bacteria; p__Bacteroidetes; c__Bacteroidia; o__Bacteroidales; f__Rikenellaceae; g__Alistipes; s__indistinctus
5c82dc47435864e490625ae01151570e	0.801226676	-1.196467707	k__Bacteria; p__Firmicutes; c__Clostridia; o__Clostridiales
df15aa00cffdc5235078831e54deb6db	0.704648316	-1.196171591	k__Bacteria; p__Firmicutes; c__Clostridia; o__Clostridiales
2628a2fe13ec39536b74fad867bec67d	-1.127480447	-1.170559952	k__Bacteria; p__Firmicutes; c__Clostridia; o__Clostridiales; f__Ruminococcaceae; g__Anaerotruncus; s__
fb9932508b23e61c5b28a7a1f84ff65a	-0.766754567	-1.15657861	k__Bacteria; p__Firmicutes; c__Clostridia; o__Clostridiales; f__Lachnospiraceae
72040df88c6e87af68cce267f9fc9b91	-1.396510064	-1.151638816	k__Bacteria; p__Proteobacteria; c__Deltaproteobacteria; o__Desulfovibrionales; f__Desulfovibrionaceae; g__Bilophila; s__
f76444e08a959e121abde93d2207186f	-0.115006387	-1.13906009	k__Bacteria; p__Proteobacteria; c__Betaproteobacteria; o__Burkholderiales; f__Alcaligenaceae; g__Sutterella; s__
a0a476401d529ced24788bff2ffd4515	-1.984808385	-1.137727807	k__Bacteria; p__Firmicutes; c__Clostridia; o__Clostridiales; f__Lachnospiraceae; g__Blautia
dfa48c96dd731ab213a1a57f885c95ac	-0.837474763	-1.111580203	k__Bacteria; p__Firmicutes; c__Clostridia; o__Clostridiales; f__Lachnospiraceae
bba2072bd8d23a0347d920f1aea6012c	-1.731227815	-1.091080258	k__Bacteria; p__Bacteroidetes; c__Bacteroidia; o__Bacteroidales; f__[Odoribacteraceae]; g__Butyricimonas; s__
14f9cc8ecdda2728b7966b31a87b17a4	-0.520385444	-1.079577992	k__Bacteria; p__Firmicutes; c__Clostridia; o__Clostridiales; f__Lachnospiraceae; g__Lachnobacterium; s__
d32f24bb89dd024a38ac0a7a5962c771	1.556677044	-1.07779033	k__Bacteria; p__Firmicutes; c__Clostridia; o__Clostridiales; f__Lachnospiraceae; g__Lachnospira; s__
504b5f0d2545c712940d4b9772a89711	1.418703378	-1.07700927	k__Bacteria; p__Firmicutes; c__Clostridia; o__Clostridiales
ef24efd75bdfb7999234e43dd6f29d7d	0.582718194	-1.070610116	k__Bacteria; p__Firmicutes; c__Clostridia; o__Clostridiales; f__Ruminococcaceae
4591b36f515c6873d3a105b9ae2bc684	-1.568580091	-1.05992038	k__Bacteria; p__Firmicutes; c__Clostridia; o__Clostridiales; f__Lachnospiraceae
6384780b6f2772d3125d3179601767fb	-1.351982057	-1.048755953	k__Bacteria; p__Firmicutes; c__Clostridia; o__Clostridiales; f__Lachnospiraceae; g__Clostridium; s__citroniae
26c9f4f844da09bdd5c1603be193969c	1.274684251	-1.046117375	k__Bacteria; p__Bacteroidetes; c__Bacteroidia; o__Bacteroidales; f__Porphyromonadaceae; g__Parabacteroides
8e200bf21884bda63c42e6df95e39e8f	-1.901600301	-1.004916022	k__Bacteria; p__Firmicutes; c__Clostridia; o__Clostridiales; f__Lachnospiraceae
4be5ec3f2050a6bc7db8d4b18e74afc5	2.786756635	-1.002435515	k__Bacteria; p__Firmicutes; c__Clostridia; o__Clostridiales; f__Lachnospiraceae; g__Roseburia; s__faecis
89170e396d8cb8428b49bdd635b91272	0.467938006	-0.997022221	k__Bacteria; p__Proteobacteria; c__Betaproteobacteria; o__Burkholderiales; f__Alcaligenaceae; g__Sutterella; s__
0eabee792a3518e67acfd8a1aec7650e	-1.945177018	-0.995146821	k__Bacteria; p__Firmicutes; c__Clostridia; o__Clostridiales; f__Lachnospiraceae
bc4b55a6e815a3321411c72f4e6ae6be	-1.613242566	-0.983501742	k__Bacteria; p__Firmicutes; c__Clostridia; o__Clostridiales; f__Ruminococcaceae; g__Ruminococcus; s__
07bf7669e42ebc17a676ae314133677b	-1.287155568	-0.975076745	k__Bacteria; p__Proteobacteria; c__Gammaproteobacteria; o__Pasteurellales; f__Pasteurellaceae; g__Aggregatibacter
c335a7c6e1e3f03c2c532274ec80a0c5	0.725093663	-0.957545827	k__Bacteria; p__Firmicutes; c__Clostridia; o__Clostridiales; f__Lachnospiraceae; g__Roseburia; s__
cb4e8d52f14846eb8f5efc2bdff31d96	-1.441568792	-0.943266938	k__Bacteria; p__Firmicutes; c__Clostridia; o__Clostridiales; f__Ruminococcaceae
d048172b7b49bf1669197f10389312cd	0.202704251	-0.920501063	k__Bacteria; p__Firmicutes; c__Clostridia; o__Clostridiales; f__Ruminococcaceae; g__Ruminococcus; s__
e49b400d5dd0a8b8e55d2ebcdef3a5a4	-0.589621484	-0.905596564	k__Bacteria; p__Proteobacteria; c__Deltaproteobacteria; o__Desulfovibrionales; f__Desulfovibrionaceae; g__Bilophila; s__
b8eddd1fb3af049687c92adf7eb1efab	1.264837802	-0.885078738	k__Bacteria; p__Firmicutes; c__Clostridia; o__Clostridiales; f__Ruminococcaceae; g__Oscillospira; s__

b5273023863d8556eae399fbe127bfa1	1.104108393	-0.868701766	k__Bacteria; p__Firmicutes; c__Clostridia; o__Clostridiales; f__Ruminococcaceae; g__Ruminococcus; s__
c4187ca9212063e21c3b7c7b4de15927	1.349270642	-0.865184853	k__Bacteria; p__Firmicutes; c__Clostridia; o__Clostridiales; f__Ruminococcaceae
c4995645e0c1545b0e1620144d03772b	2.970455408	-0.861420701	k__Bacteria; p__Firmicutes; c__Clostridia; o__Clostridiales; f__Ruminococcaceae
23fed68c6c76ab10ba1be8a43e9176e7	3.691843599	-0.857554267	k__Bacteria; p__Firmicutes; c__Clostridia; o__Clostridiales; f__Ruminococcaceae; g__Faecalibacterium; s__prausnitzii
935636058964e21caeffa5f7b9611e09	1.374363482	-0.857278893	k__Bacteria; p__Firmicutes; c__Clostridia; o__Clostridiales; f__Lachnospiraceae; g__Lachnospira; s__
3cb45e869029068a0939766280fcd1b2	-1.342800081	-0.832991193	k__Bacteria; p__Firmicutes; c__Clostridia; o__Clostridiales; f__Lachnospiraceae; g__Lachnospira; s__
b626841d59de09e32958b7bb7080e06b	-1.558271348	-0.799264262	k__Bacteria; p__Firmicutes; c__Clostridia; o__Clostridiales; f__Ruminococcaceae; g__Oscillospira; s__
fcdf3e1e3fd860c9a76a22fe6da59882	-0.975329339	-0.798713277	k__Bacteria; p__Bacteroidetes; c__Bacteroidia; o__Bacteroidales; f__Prevotellaceae; g__Prevotella; s__
98ff1f2b1008b5e0971997cd5070fc03	0.406484187	-0.780671427	k__Bacteria; p__Firmicutes; c__Clostridia; o__Clostridiales; f__Ruminococcaceae; g__Ruminococcus; s__
8a1666ef29dadb7bf02e29aaea442cd1	-0.403259933	-0.77826793	k__Bacteria; p__Firmicutes; c__Clostridia; o__Clostridiales; f__Lachnospiraceae
e47a63686b619f67783f9b9aa52b86bf	3.481671613	-0.761317084	k__Bacteria; p__Bacteroidetes; c__Bacteroidia; o__Bacteroidales; f__Rikenellaceae; g__Alistipes; s__putredinis
8111edbcdbb3921142e30e5d14b6d311	1.288578809	-0.759987543	k__Bacteria; p__Firmicutes; c__Clostridia; o__Clostridiales; f__Ruminococcaceae
ec54027279e7e3776bf29227919b98d5	0.174819052	-0.756793926	k__Bacteria; p__Firmicutes; c__Clostridia; o__Clostridiales; f__Ruminococcaceae
7e277c0d03cf34b02f23a82aacb1d560	-0.804459035	-0.742608378	k__Bacteria; p__Bacteroidetes; c__Bacteroidia; o__Bacteroidales; f__[Odoribacteraceae]; g__Odoribacter; s__
17e769c380468fab914321be7723b865	3.297247589	-0.739173005	k__Bacteria; p__Bacteroidetes; c__Bacteroidia; o__Bacteroidales; f__Bacteroidaceae; g__Bacteroides; s__coprophilus
0437c9910becc153a3d7838fd8eaa64b	0.256977618	-0.719067285	k__Bacteria; p__Firmicutes; c__Clostridia; o__Clostridiales; f__Ruminococcaceae; g__Butyricicoccus; s__pullicaecorum
1bbe672253a9a19978b7163262e6db45	1.886092603	-0.710349868	k__Bacteria; p__Bacteroidetes; c__Bacteroidia; o__Bacteroidales; f__Prevotellaceae; g__Prevotella; s__stercorea
75622b8ee0f6a2b8a796bbfd264ca9fa	1.178019345	-0.674682686	k__Bacteria; p__Firmicutes; c__Clostridia; o__Clostridiales; f__Lachnospiraceae; g__Anaerostipes; s__
3ea67969a01ec419ec1d4784d379ad1d	1.244925559	-0.661822508	k__Bacteria; p__Bacteroidetes; c__Bacteroidia; o__Bacteroidales; f__[Odoribacteraceae]; g__Odoribacter; s__
a138db10fd3b444fd9eaca5c18ed0381	1.991260588	-0.646299193	k__Bacteria; p__Bacteroidetes; c__Bacteroidia; o__Bacteroidales; f__[Paraprevotellaceae]; g__Paraprevotella; s__
572c5229c641b2dc5124ea44e4edef4c	0.547756017	-0.62600405	k__Bacteria; p__Bacteroidetes; c__Bacteroidia; o__Bacteroidales; f__Rikenellaceae; g__Alistipes; s__indistinctus
37dac50f454a3cf754e83790eee77108	0.637056887	-0.61267061	k__Bacteria; p__Bacteroidetes; c__Bacteroidia; o__Bacteroidales; f__[Paraprevotellaceae]; g__Paraprevotella; s__
d6777df424c4e02b9aa8c74ba8f0a54d	-1.69064945	-0.604135702	k__Bacteria; p__Bacteroidetes; c__Bacteroidia; o__Bacteroidales; f__[Odoribacteraceae]; g__Butyricimonas; s__
d18751daa1bf5ff843bab70836374d5f	-1.109190881	-0.596416543	k__Bacteria; p__Firmicutes; c__Clostridia; o__Clostridiales
35ffdd51464e2c68179717e5334a1d7e	0.92274195	-0.583589385	k__Bacteria; p__Bacteroidetes; c__Bacteroidia; o__Bacteroidales; f__Bacteroidaceae; g__Bacteroides; s__fragilis
2cad9fc22fd99d0c6d900977f522b2d9	5.039157212	-0.582421014	k__Bacteria; p__Bacteroidetes; c__Bacteroidia; o__Bacteroidales; f__Bacteroidaceae; g__Bacteroides; s__plebeius
7082034a5eec4c381d09cd380594242e	-0.403632819	-0.568975041	k__Bacteria; p__Firmicutes; c__Clostridia; o__Clostridiales; f__Lachnospiraceae; g__Blautia; s__
e372b34ee2ffaa78a8f7ef65c0b2f153	-0.864546239	-0.563465069	k__Bacteria; p__Firmicutes; c__Clostridia; o__Clostridiales; f__Ruminococcaceae; g__Oscillospira; s__

5f2d589aeca05cfa41265dd5e23603b4	0.821825326	-0.562213848	k__Bacteria; p__Firmicutes; c__Clostridia; o__Clostridiales; f__Ruminococcaceae; g__Ruminococcus; s__callidus
ee293984c0110b2eeceb8427fdf448fb	1.955739796	-0.56019337	k__Bacteria; p__Firmicutes; c__Clostridia; o__Clostridiales; f__Lachnospiraceae; g__Blautia; s__obeum
ef28677a61a54aec2ee09eb7958257d6	-1.107406556	-0.559170911	k__Bacteria; p__Firmicutes; c__Clostridia; o__Clostridiales; f__Lachnospiraceae
c838eb5b3b3d9fd0dfecdf75547c3c63	0.334312499	-0.557606886	k__Bacteria; p__Firmicutes; c__Clostridia; o__Clostridiales
574ab9c17692ffd001643c930f6895f5	2.056369841	-0.556153128	k__Bacteria; p__Bacteroidetes; c__Bacteroidia; o__Bacteroidales; f__Bacteroidaceae; g__Bacteroides; s__uniformis
07dae4672402022ad477f7aabdebb7bb	-0.492790401	-0.555801342	k__Bacteria; p__Firmicutes; c__Clostridia; o__Clostridiales; f__[Mogibacteriaceae]; g__Mogibacterium; s__
ae734c4d175f73fc729b068e4305b721	-1.73074907	-0.549816201	k__Bacteria; p__Firmicutes; c__Clostridia; o__Clostridiales; f__Lachnospiraceae; g__[Ruminococcus]; s__gnavus
b15165107bf1c1c029df56aacc13ecf3	1.170283616	-0.54516227	k__Bacteria; p__Firmicutes; c__Clostridia; o__Clostridiales; f__; g__; s__
22cda660a06046e7a39c98b2432c80c5	-0.176474988	-0.543530653	k__Bacteria; p__Bacteroidetes; c__Bacteroidia; o__Bacteroidales; f__[Odoribacteraceae]; g__Butyricimonas; s__
8cb3c10395e199b1b656b6b94a25957c	-2.53307575	-0.54028661	k__Bacteria; p__Firmicutes; c__Clostridia; o__Clostridiales; f__Lachnospiraceae; g__Blautia; s__
fc2eeb02cc0e0e7f598b88912ae6c891	0.8501845	-0.537221382	k__Bacteria; p__Firmicutes; c__Clostridia; o__Clostridiales; f__Lachnospiraceae; g__Coprococcus; s__catus
b944e446e87fa7a72fe2cccb95deee14	-2.553960263	-0.536252568	k__Bacteria; p__Actinobacteria; c__Actinobacteria; o__Actinomycetales; f__Micrococcaceae; g__Rothia; s__mucilaginosa
e318bc2a156c4c88fba4efa0a0ab403a	0.135550797	-0.534122536	k__Bacteria; p__Firmicutes; c__Clostridia; o__Clostridiales; f__Veillonellaceae; g__Dialister; s__
11ac06f483b516aa29d898de98501615	-1.955034196	-0.52576072	k__Bacteria; p__Firmicutes; c__Clostridia; o__Clostridiales; f__Ruminococcaceae
9ef6efd0e49ef5e1efe3613b0e427312	2.43924886	-0.524779389	k__Bacteria; p__Firmicutes; c__Clostridia; o__Clostridiales; f__Lachnospiraceae; g__Roseburia; s__
a070c2e394db428a514dfa385c502b8e	3.251838997	-0.51393647	k__Bacteria; p__Firmicutes; c__Clostridia; o__Clostridiales; f__Ruminococcaceae; g__; s__
91929298890f00ecf28f27d94735bfea	-1.811448514	-0.49357302	k__Bacteria; p__Firmicutes; c__Clostridia; o__Clostridiales
fd2130b6a8a1db45c0f5381012b2e5e7	1.493623674	-0.486909578	k__Bacteria; p__Firmicutes; c__Clostridia; o__Clostridiales; f__; g__; s__
5898ec3a80ad76122c711774a3be2994	0.9033373	-0.48343272	k__Bacteria; p__Firmicutes; c__Clostridia; o__Clostridiales; f__Ruminococcaceae
619f64f2bf103286f4f70bfd89500ed4	0.844395936	-0.482260297	k__Bacteria; p__Bacteroidetes; c__Bacteroidia; o__Bacteroidales; f__Rikenellaceae; g__Alistipes; s__finegoldii
b6917fd2056dd756f7383cfdd246c7cf	-2.130114495	-0.473053644	k__Bacteria; p__Bacteroidetes; c__Bacteroidia; o__Bacteroidales; f__Porphyromonadaceae; g__Parabacteroides; s__distasonis
b20c095fd654b84cebdbfe4faa0a1409	0.558942139	-0.471830199	k__Bacteria; p__Firmicutes; c__Clostridia; o__Clostridiales; f__Lachnospiraceae
4119ee9eb78e6baa92065d886a5e24ac	-1.596249521	-0.468796084	k__Bacteria; p__Firmicutes; c__Clostridia; o__Clostridiales; f__[Mogibacteriaceae]; g__; s__
81498438a4a99ab2ba5e04ffba72748e	-0.853424489	-0.443799327	k__Bacteria; p__Firmicutes; c__Bacilli; o__Lactobacillales; f__Streptococcaceae; g__Lactococcus; s__garvieae
cb6e7abe25ee6b90ba4154f0aa33e5f6	-2.361531675	-0.436762521	k__Bacteria; p__Bacteroidetes; c__Bacteroidia; o__Bacteroidales; f__Porphyromonadaceae; g__Parabacteroides; s__distasonis
e17d964cd0fd8c2ddbd1dacfa8b536e3	3.438959441	-0.422434042	k__Bacteria; p__Bacteroidetes; c__Bacteroidia; o__Bacteroidales; f__Porphyromonadaceae; g__Parabacteroides; s__
8f781386e4c35571502a9af87fb53051	-2.109330117	-0.419979522	k__Bacteria; p__Firmicutes; c__Clostridia; o__Clostridiales; f__Veillonellaceae; g__Dialister; s__
8b8b93b27e9c6cc58aacb0bf38248700	-1.569257199	-0.412162373	k__Bacteria; p__Firmicutes; c__Clostridia; o__Clostridiales; f__Lachnospiraceae

7e048b0e0c32d2f839e9f2098a4ebb35	-1.321202695	-0.404085586	k__Bacteria; p__Firmicutes; c__Clostridia; o__Clostridiales; f__Lachnospiraceae
a7283edda8770d12b4e25d4dcce112c4	-0.990581453	-0.387484739	k__Bacteria; p__Firmicutes; c__Clostridia; o__Clostridiales; f__Ruminococcaceae; g__Oscillospira; s__
157eb8623615df51a717c3a9b85e41b6	-1.248544633	-0.359877298	k__Bacteria; p__Firmicutes; c__Clostridia; o__Clostridiales; f__Lachnospiraceae
dffa04ca171f459056b9466d7acfe601	1.294315636	-0.357759307	k__Bacteria; p__Firmicutes; c__Clostridia; o__Clostridiales; f__Lachnospiraceae
1d38c3d3e21b0567dac79cc36187431a	3.732091666	-0.354741404	k__Bacteria; p__Bacteroidetes; c__Bacteroidia; o__Bacteroidales; f__[Paraprevotellaceae]; g__[Prevotella]; s__
f90e88c52df1bc147bfe9705270cb88f	-1.431632936	-0.3336323	k__Bacteria; p__Bacteroidetes; c__Bacteroidia; o__Bacteroidales; f__Prevotellaceae; g__Prevotella; s__
ccd67c5913cf31adeecd1e3f741054c8	-1.26677078	-0.331914017	k__Bacteria; p__Bacteroidetes; c__Bacteroidia; o__Bacteroidales; f__Porphyromonadaceae; g__Parabacteroides
10541b6401f0ea0b38e3fcd6265d3c86	2.547402561	-0.321590493	k__Bacteria; p__Firmicutes; c__Erysipelotrichi; o__Erysipelotrichales; f__Erysipelotrichaceae; g__[Eubacterium]; s__biforme
ddecb16f6a6e5aa3dd83029b491c9958	-0.957195222	-0.317064593	k__Bacteria; p__Bacteroidetes; c__Bacteroidia; o__Bacteroidales; f__Prevotellaceae; g__Prevotella; s__
b953ac3c6973b5a557774d4b2d005d48	1.931995213	-0.312488268	k__Bacteria; p__Firmicutes; c__Erysipelotrichi; o__Erysipelotrichales; f__Erysipelotrichaceae; g__Catenibacterium; s__
b3bd0d387d67ca01fe3197f5bf66b032	0.406752169	-0.311660359	k__Bacteria; p__Firmicutes; c__Clostridia; o__Clostridiales; f__Lachnospiraceae; g__Coprococcus; s__
fc02af89d869043b296114729009a5b0	3.074471295	-0.30764837	k__Bacteria; p__Firmicutes; c__Clostridia; o__Clostridiales; f__Ruminococcaceae; g__Ruminococcus; s__bromii
fb798da6858b8b3f34077164a4ec9315	1.275274337	-0.299746821	k__Bacteria; p__Firmicutes; c__Clostridia; o__Clostridiales; f__Lachnospiraceae
eb61cae65bc6cdd2440323bbf603ba5c	0.916879714	-0.296408007	k__Bacteria; p__Firmicutes; c__Clostridia; o__Clostridiales; f__Lachnospiraceae
58c9d27620c4a8f749c75f945fce11a0	-2.250544488	-0.296163986	k__Bacteria; p__Firmicutes; c__Clostridia; o__Clostridiales; f__Lachnospiraceae; g__Coprococcus; s__
7c93315259ad14510b6e9cae0670a048	-0.192608535	-0.287189434	k__Bacteria; p__Firmicutes; c__Clostridia; o__Clostridiales; f__Ruminococcaceae; g__Oscillospira; s__
08ce8cd4ee7a48f9342c9931f38d8328	-1.189579427	-0.277992556	k__Bacteria; p__Bacteroidetes; c__Bacteroidia; o__Bacteroidales; f__[Paraprevotellaceae]; g__Paraprevotella; s__
3ada9b89ff4552792c8460298ee66b64	-0.975978315	-0.275488446	k__Bacteria; p__Firmicutes; c__Clostridia; o__Clostridiales; f__Lachnospiraceae; g__Clostridium; s__colinum
ca8848e910de604b4150bd50e6a5cf51	2.257517159	-0.275128672	k__Bacteria; p__Bacteroidetes; c__Bacteroidia; o__Bacteroidales; f__[Paraprevotellaceae]; g__Paraprevotella; s__
a1a300c872047a7d8b6b294b17cfb63b	0.518731416	-0.274780104	k__Bacteria; p__Firmicutes; c__Clostridia; o__Clostridiales; f__Ruminococcaceae
689b5301572a169a6995d77a0be03430	-2.153754651	-0.258834908	k__Bacteria; p__Bacteroidetes; c__Bacteroidia; o__Bacteroidales; f__Bacteroidaceae; g__Bacteroides; s__
e3ca4f48b9547eaf8ef9d4d3ea4ff165	-1.472500741	-0.257237504	k__Bacteria; p__Firmicutes; c__Clostridia; o__Clostridiales; f__Veillonellaceae; g__Phascolarctobacterium; s__
2d34c22edce4b1f2d8a5228ad78f8ea8	4.311037302	-0.252674887	k__Bacteria; p__Firmicutes; c__Clostridia; o__Clostridiales; f__Lachnospiraceae; g__Roseburia; s__faecis
2fe1d8c841ad33039a4a46d652c59698	-2.089767396	-0.249933431	k__Bacteria; p__Firmicutes; c__Clostridia; o__Clostridiales; f__Lachnospiraceae
d9681bf8373e2635323f1ca0cc434ecf	-1.646029412	-0.243929455	k__Bacteria; p__Firmicutes; c__Clostridia; o__Clostridiales; f__Lachnospiraceae
fcd2073fc9628403d2dc4aa2b66b64a5	-0.569961488	-0.238557289	k__Bacteria; p__Firmicutes; c__Clostridia; o__Clostridiales; f__Lachnospiraceae
d276e683f00aadfd097c1a0d7439c487	-1.12160629	-0.235372732	k__Bacteria; p__Firmicutes; c__Clostridia; o__Clostridiales; f__Lachnospiraceae

c5465580c4c4cdd15a78de5a240dbd4e	0.656341851	-0.216388414	k__Bacteria; p__Firmicutes; c__Clostridia; o__Clostridiales; f__Lachnospiraceae
5b623d235d84023ed2be03980e1889cf	-1.604012429	-0.215462992	k__Bacteria; p__Firmicutes; c__Clostridia; o__Clostridiales; f__Lachnospiraceae; g__Clostridium
2cdb259b754c2db622ed9fb5a6517a37	-1.740472257	-0.20137615	k__Bacteria; p__Bacteroidetes; c__Bacteroidia; o__Bacteroidales; f__Porphyromonadaceae; g__Parabacteroides; s__distasonis
a0b7d83fb64749f9a4b15b3728425b97	1.253515542	-0.192537854	k__Bacteria; p__Firmicutes; c__Clostridia; o__Clostridiales; f__Lachnospiraceae; g__Dorea; s__
4d77a61ec39a89bc7965dbc59d2a724d	-0.834155976	-0.183689306	k__Bacteria; p__Firmicutes; c__Clostridia; o__Clostridiales; f__Ruminococcaceae; g__Butyricicoccus; s__pullicaecorum
f54512749b97be9497134dc28c0af837	0.643597424	-0.183186839	k__Bacteria; p__Firmicutes; c__Clostridia; o__Clostridiales; f__Lachnospiraceae
63b26504f32377cd78d6068bffb86b9a	3.78776452	-0.183101127	k__Bacteria; p__Bacteroidetes; c__Bacteroidia; o__Bacteroidales; f__Bacteroidaceae; g__Bacteroides; s__uniformis
6bade26b26bfecfc551333e80ea07501	-1.977188527	-0.177935312	k__Bacteria; p__Firmicutes; c__Clostridia; o__Clostridiales; f__Ruminococcaceae; g__Oscillospira; s__
143df73c0bb17e89fbd72290a8e569e2	-1.838716924	-0.170176814	k__Bacteria; p__Firmicutes; c__Clostridia; o__Clostridiales; f__Ruminococcaceae
f66417ef7725f585f74939928b81650d	-2.411234796	-0.166102359	k__Bacteria; p__Firmicutes; c__Clostridia; o__Clostridiales
f79d253cad27c0e1a9fd63f32a9e337a	-1.614340722	-0.161562989	k__Bacteria; p__Firmicutes; c__Clostridia; o__Clostridiales; f__Lachnospiraceae
c3bdda568b2c1580d5cce7407ef43909	3.980299413	-0.154612611	k__Bacteria; p__Firmicutes; c__Clostridia; o__Clostridiales; f__Ruminococcaceae; g__Faecalibacterium; s__prausnitzii
a3bf9252a3063e2844dcade5e4192e50	1.720216573	-0.14418144	k__Bacteria; p__Firmicutes; c__Clostridia; o__Clostridiales; f__Lachnospiraceae; g__Coprococcus; s__
03af966ff07ddef2b87da992b85b600b	2.774211169	-0.143889854	k__Bacteria; p__Bacteroidetes; c__Bacteroidia; o__Bacteroidales; f__Bacteroidaceae; g__Bacteroides; s__ovatus
aad4ab75d4049ba9bc82a856845fbeeb	1.290903867	-0.100337217	k__Bacteria; p__Firmicutes; c__Clostridia; o__Clostridiales; f__Ruminococcaceae; g__Butyricicoccus; s__pullicaecorum
4bc7315a09f4753c6454ebc80bef05af	0.615949691	-0.095386098	k__Bacteria; p__Firmicutes; c__Clostridia; o__Clostridiales; f__Lachnospiraceae; g__Coprococcus; s__
c1e6a3b26cc7d12c8f7e22ebb092e001	3.426287624	-0.095042179	k__Bacteria; p__Bacteroidetes; c__Bacteroidia; o__Bacteroidales; f__Bacteroidaceae; g__Bacteroides; s__uniformis
9e1d23e474a9f1e4e10387a23f601dde	1.136749327	-0.085648368	k__Bacteria; p__Firmicutes; c__Clostridia; o__Clostridiales; f__Ruminococcaceae; g__Oscillospira; s__
5a0ac2e0b9d96cd805f282a415d7a385	-0.098785579	-0.08448107	k__Bacteria; p__Proteobacteria; c__Deltaproteobacteria; o__Desulfovibrionales; f__Desulfovibrionaceae; g__Bilophila; s__
694d61b8ec78349749c8b6ea59938e0b	0.718141616	-0.083645771	k__Bacteria; p__Firmicutes; c__Clostridia; o__Clostridiales; f__Lachnospiraceae
7d17d197d7978be0b6684209ce257e20	2.538016379	-0.074927638	k__Bacteria; p__Proteobacteria; c__Betaproteobacteria; o__Burkholderiales; f__Alcaligenaceae; g__Sutterella; s__
84e9e2afcdae1240b3ce3267067e1879	-0.800697744	-0.07465298	k__Bacteria; p__Firmicutes; c__Erysipelotrichi; o__Erysipelotrichales; f__Erysipelotrichaceae; g__Holdemania; s__
d5c7d97e6f4f5789d574d321dcca0992	2.682467997	-0.070620606	k__Bacteria; p__Firmicutes; c__Clostridia; o__Clostridiales; f__Lachnospiraceae
0d8b9177d01f328c2f5d5efa4c2acbc5	1.178683579	-0.070112655	k__Bacteria; p__Bacteroidetes; c__Bacteroidia; o__Bacteroidales; f__Porphyromonadaceae; g__Parabacteroides; s__
4bf3198c78397be5af0b7325d20558de	5.651480735	-0.058446238	k__Bacteria; p__Bacteroidetes; c__Bacteroidia; o__Bacteroidales; f__Prevotellaceae; g__Prevotella; s__copri
0bb34d08155d892896cec6aef8c49449	1.575801075	-0.055005977	k__Bacteria; p__Fusobacteria; c__Fusobacteriia; o__Fusobacteriales; f__Fusobacteriaceae; g__Fusobacterium; s__
3e8fee153cbee59353604d9f638026af	-2.559956968	-0.054741333	k__Bacteria; p__Bacteroidetes; c__Bacteroidia; o__Bacteroidales; f__Porphyromonadaceae; g__Parabacteroides; s__distasonis
b4e6bfdac717a87fd200d75534fdf5d3	-1.385751664	-0.052868555	k__Bacteria; p__Bacteroidetes; c__Bacteroidia; o__Bacteroidales; f__[Barnesiellaceae]; g__; s__

dc2721103659fe9f1d3ead56a11df243	2.442636431	-0.050793002	k__Bacteria; p__Firmicutes; c__Clostridia; o__Clostridiales; f__Lachnospiraceae; g__Blautia; s__
e7c78293d64fdce36eee64193ccc346a	-0.011269986	-0.042612503	k__Bacteria; p__Bacteroidetes; c__Bacteroidia; o__Bacteroidales; f__Bacteroidaceae; g__Bacteroides
f7ae6b2c56937893ef465d6e830b22a0	1.865330637	-0.029782126	k__Bacteria; p__Firmicutes; c__Clostridia; o__Clostridiales; f__Ruminococcaceae; g__Ruminococcus; s__bromii
429799da351a899f9d472ef9e28c1028	-1.290197313	-0.016802142	k__Bacteria; p__Firmicutes; c__Clostridia; o__Clostridiales; f__; g__; s__
ebbe8017bca36f0a3b02532a2b4ed0d2	1.346596062	-0.009095857	k__Bacteria; p__Firmicutes; c__Clostridia; o__Clostridiales; f__Lachnospiraceae
8468e308e3ff23c41059c416ba2b2676	1.573742688	0.000233104	k__Bacteria; p__Firmicutes; c__Clostridia; o__Clostridiales; f__Veillonellaceae; g__Phascolarctobacterium; s__
d4c69fc5a8668eaa728643f54865842c	-1.454048097	0.001147082	k__Bacteria; p__Bacteroidetes; c__Bacteroidia; o__Bacteroidales; f__Bacteroidaceae; g__Bacteroides; s__
e5450b2648c26b644c8c5df0535bd4c2	-0.085249841	0.002590587	k__Bacteria; p__Firmicutes; c__Clostridia; o__Clostridiales; f__Veillonellaceae; g__Acidaminococcus; s__
457ee45e6a2eda370c854acc25056938	-0.858222902	0.006500532	k__Bacteria; p__Firmicutes; c__Clostridia; o__Clostridiales; f__Lachnospiraceae; g__Blautia
c9ea71f39bda8752713c8e90dff2b875	1.809350074	0.01208215	k__Bacteria; p__Firmicutes; c__Clostridia; o__Clostridiales; f__Lachnospiraceae; g__Coprococcus; s__
b677c6c95bbf603529595ed92876ef37	0.374360621	0.012822916	k__Bacteria; p__Firmicutes; c__Erysipelotrichi; o__Erysipelotrichales; f__Erysipelotrichaceae; g__[Eubacterium]; s__biforme
d8eb518d96bf708e8353e78d62a39303	-0.330486715	0.015244176	k__Bacteria; p__Bacteroidetes; c__Bacteroidia; o__Bacteroidales; f__[Odoribacteraceae]; g__Butyricimonas; s
1b158b8b2922d4fcad5d9cea607cbb7d	5.755636275	0.023233821	k__Bacteria; p__Proteobacteria; c__Gammaproteobacteria; o__Enterobacteriales; f__Enterobacteriaceae
ea83b86e362b930cd09e51487819f953	-1.439939916	0.023950507	k__Bacteria; p__Bacteroidetes; c__Bacteroidia; o__Bacteroidales; f__Porphyromonadaceae; g__Parabacteroides; s
bde76e426d074e3859d6231de33f6693	-0.709143102	0.025910904	k__Bacteria; p__Firmicutes; c__Clostridia; o__Clostridiales; f__Lachnospiraceae; g__Blautia; s__producta
6ab375a376f4b496b450e1142ac395e1	-2.271319806	0.030296614	k__Bacteria; p__Firmicutes; c__Clostridia; o__Clostridiales; f__Lachnospiraceae
61599d57af1407a8086462e4bb761b98	-3.038378179	0.034313252	k__Bacteria; p__Bacteroidetes; c__Bacteroidia; o__Bacteroidales; f__Bacteroidaceae; g__Bacteroides; s__
51e441cbdcc80da0656e82293ae160b5	2.922797025	0.037486722	k__Bacteria; p__Bacteroidetes; c__Bacteroidia; o__Bacteroidales; f__Bacteroidaceae; g__Bacteroides
e9703768a50971c05b34b102810fd761	-0.516317546	0.03759115	k__Bacteria; p__Firmicutes; c__Clostridia; o__Clostridiales; f__Lachnospiraceae; g__Blautia; s__
540414207acda390b7e8a3bf245b61c6	-1.836528718	0.039565971	k__Bacteria; p__Firmicutes; c__Clostridia; o__Clostridiales; f__Ruminococcaceae; g__Oscillospira; s__
ee8c3fb02b1233997432564a8ba62319	-0.811786592	0.058425	k__Bacteria; p__Bacteroidetes; c__Bacteroidia; o__Bacteroidales; f__Prevotellaceae; g__Prevotella; s__
554c761996ebab999befda1b695fd81d	2.68649149	0.058581045	k__Bacteria; p__Actinobacteria; c__Actinobacteria; o__Bifidobacteriales; f__Bifidobacteriaceae; g__Bifidobacterium; s__adolescentis
710831ae76a15dbe4d17e50fb02e276f	-1.99329704	0.063173106	k__Bacteria; p__Proteobacteria; c__Deltaproteobacteria; o__Desulfovibrionales; f__Desulfovibrionaceae; g__; s__
a67cb378efc39872e25df8467bd8616e	3.835301817	0.069361975	k__Bacteria; p__Bacteroidetes; c__Bacteroidia; o__Bacteroidales; f__Prevotellaceae; g__Prevotella; s__copri
dad9c1aff4e6d7faab64199863616bc7	1.363170207	0.077270558	k__Bacteria; p__Bacteroidetes; c__Bacteroidia; o__Bacteroidales; f__Prevotellaceae; g__Prevotella; s__copri
588b47dbaad314c687436406aca37192	-1.275226056	0.086306264	k__Bacteria; p__Firmicutes; c__Clostridia; o__Clostridiales; f__Ruminococcaceae
a3f36ef32153f2fc2aaeac2feb23777f	3.84701705	0.089322736	k__Bacteria; p__Firmicutes; c__Clostridia; o__Clostridiales; f__Ruminococcaceae; g__Faecalibacterium; s__prausnitzii

668fdb718997fc1589c7817655d4bb5f	5.675806344	0.099299719	k__Bacteria; p__Bacteroidetes; c__Bacteroidia; o__Bacteroidales; f__Bacteroidaceae; g__Bacteroides; s__
0fed1d1744ec3a03cf7db18a94d7643c	1.445683062	0.101856282	k__Bacteria; p__Bacteroidetes; c__Bacteroidia; o__Bacteroidales; f__Prevotellaceae; g__Prevotella; s__stercorea
aed3f59201e3b9d21858f36557f42a80	3.675402016	0.107067993	k__Bacteria; p__Firmicutes; c__Clostridia; o__Clostridiales; f__Ruminococcaceae; g__Gemmiger; s__formicilis
f2e4355abb553aed0f53a97b59f2acf2	1.564371527	0.113893678	k__Bacteria; p__Proteobacteria; c__Gammaproteobacteria; o__Enterobacteriales; f__Enterobacteriaceae
d5f386f1a19e3146f461548e62256919	-0.873857438	0.115834763	k__Bacteria; p__Proteobacteria; c__Deltaproteobacteria; o__Desulfovibrionales; f__Desulfovibrionaceae; g__; s__
d3247c936f8f4735909a8526ebf2f49e	-0.22477597	0.118094852	k__Bacteria; p__Firmicutes; c__Clostridia; o__Clostridiales; f__Veillonellaceae; g__Veillonella; s__parvula
520c77820886daeb8cf0d6497cfb1344	1.602885902	0.123206904	k__Bacteria; p__Firmicutes; c__Clostridia; o__Clostridiales; f__Veillonellaceae; g__Dialister; s__
ac7e3a431bf7dec4fe9a731bd1976c2a	-2.353532254	0.126424601	k__Bacteria; p__Bacteroidetes; c__Bacteroidia; o__Bacteroidales; f__Bacteroidaceae; g__Bacteroides; s__
0c0c2430c2d825acdc22a2ffdef7e079	-1.127451837	0.13670068	k__Bacteria; p__Firmicutes; c__Erysipelotrichi; o__Erysipelotrichales; f__Erysipelotrichaceae; g__[Eubacterium]; s__biforme
a531f9e2efa1d90dd159b241ce2e9a6e	0.90181619	0.136709621	k__Bacteria; p__Firmicutes; c__Clostridia; o__Clostridiales
ac04fef6eb13ddf89756a0af35b512fe	-0.745392739	0.1563118	k__Bacteria; p__Firmicutes; c__Clostridia; o__Clostridiales; f__Lachnospiraceae
d49b8a96c69e00c79acbc5ef2d25f807	-3.03728956	0.156454375	k__Bacteria; p__Firmicutes; c__Clostridia; o__Clostridiales; f__Clostridiaceae; g__Clostridium
78314aca268ae4422bc651192fbc986d	-0.041116416	0.162228634	k__Bacteria; p__Firmicutes; c__Clostridia; o__Clostridiales; f__Lachnospiraceae
04e9151eebd435f26965d2a465f88599	2.786511123	0.169960549	k__Bacteria; p__Firmicutes; c__Clostridia; o__Clostridiales; f__Lachnospiraceae; g__Lachnospira; s__
5fdacbfd74abb06f94f87f7c539c6246	1.277517617	0.170531323	k__Bacteria; p__Bacteroidetes; c__Bacteroidia; o__Bacteroidales; f__Bacteroidaceae; g__Bacteroides
32082ec186bfa1fbe58374530e613507	-2.059655606	0.174429228	k__Bacteria; p__Bacteroidetes; c__Bacteroidia; o__Bacteroidales; f__Porphyromonadaceae; g__Parabacteroides; s__distasonis
3d0ecc54a611d9476a0db83744befa03	-1.887588441	0.177085807	k__Bacteria; p__Firmicutes; c__Clostridia; o__Clostridiales; f__Lachnospiraceae; g__[Ruminococcus]; s__gnavus
d7eb4e5c7f24ea076ecc5fa029cfbed9	-1.407928407	0.200938275	k__Bacteria; p__Firmicutes; c__Clostridia; o__Clostridiales; f__Lachnospiraceae
680d83cb233cffcc6405e08c46982042	0.292870581	0.201852014	k__Bacteria; p__Firmicutes; c__Clostridia; o__Clostridiales; f__Ruminococcaceae; g__Oscillospira; s__
4ef2b8e0ed22c2a986f53572f9e9405b	-1.314599931	0.222872307	k__Bacteria; p__Firmicutes; c__Clostridia; o__Clostridiales; f__Ruminococcaceae; g__Oscillospira; s__
44b749c0f1a0ff00e7d5274af977d9df	-1.532412469	0.247275104	k__Bacteria; p__Bacteroidetes; c__Bacteroidia; o__Bacteroidales; f__Porphyromonadaceae; g__Parabacteroides; s__
a684e731a405ce64b1669976e6658a2d	-0.363486469	0.275247922	k__Bacteria; p__Actinobacteria; c__Coriobacteriia; o__Coriobacteriales; f__Coriobacteriaceae
c0ece818b951c3d3c75a2250cfe2da48	-0.250198066	0.280711284	k__Bacteria; p__Firmicutes; c__Clostridia; o__Clostridiales; f__Lachnospiraceae; g__Clostridium; s__symbiosum
51dd453b71a4aba20d9f349601ea5203	2.022118032	0.286489119	k__Bacteria; p__Firmicutes; c__Clostridia; o__Clostridiales; f__Lachnospiraceae
5046b79b7267f5bb60cd4edb52fbe34f	1.336069167	0.30629473	k__Bacteria; p__Firmicutes; c__Clostridia; o__Clostridiales; f__; g__; s__
05a0879d203021699d913e7099da4b3e	-1.411577642	0.341363957	k__Bacteria; p__Firmicutes; c__Bacilli; o__Turicibacterales; f__Turicibacteraceae; g__Turicibacter; s__
15e25ac35ddd784dd294f0b3e424372a	-1.086087644	0.342114498	k__Bacteria; p__Firmicutes; c__Clostridia; o__Clostridiales; f__Lachnospiraceae
f4f297232da0f8d7dae7f9c432501e22	-0.410784185	0.35332041	k__Bacteria; p__Bacteroidetes; c__Bacteroidia; o__Bacteroidales; f__Porphyromonadaceae; g__Parabacteroides; s__distasonis

d9edd292dbe13f07069c196cf76da7bd	-0.313468158	0.357656946	k__Bacteria; p__Bacteroidetes; c__Bacteroidia; o__Bacteroidales; f__Bacteroidaceae; g__Bacteroides
f0607d2c57afe6bb25bd64afa9869e28	1.023952306	0.360389104	k__Bacteria; p__Firmicutes; c__Clostridia; o__Clostridiales; f__Clostridiaceae; g__; s__
9f8668eb1c5f9d9a992dd49245db090e	1.502191603	0.364226451	k__Bacteria; p__Firmicutes; c__Clostridia; o__Clostridiales; f__Lachnospiraceae; g__Blautia; s__
737e3567af27a204ac8fa1af5c611cc7	-0.084991395	0.369394173	k__Bacteria; p__Firmicutes; c__Clostridia; o__Clostridiales; f__Lachnospiraceae
e5269e32edd47bb63709e25e22a6f0ae	0.142824948	0.369492879	k__Bacteria; p__Bacteroidetes; c__Bacteroidia; o__Bacteroidales; f__Bacteroidaceae; g__Bacteroides; s__
c1dc9ad5116d96b8ed863458fc0d0aec	4.111551404	0.374339094	k__Bacteria; p__Firmicutes; c__Clostridia; o__Clostridiales; f__Lachnospiraceae; g__Blautia; s__
dadd613a5098ad82e3162201694bcc26	-1.011095941	0.376607826	k__Bacteria; p__Bacteroidetes; c__Bacteroidia; o__Bacteroidales; f__Porphyromonadaceae; g__Parabacteroides; s__distasonis
e9239125c6ba63cae1822e8da4905483	-1.898834169	0.377170434	k__Bacteria; p__Bacteroidetes; c__Bacteroidia; o__Bacteroidales; f__Porphyromonadaceae; g__Parabacteroides; s__distasonis
a7a1a93ecfcef4cb45b42307a4fa3bca	3.824510664	0.379865041	k__Bacteria; p__Bacteroidetes; c__Bacteroidia; o__Bacteroidales; f__Bacteroidaceae; g__Bacteroides; s__
5e7503d63ed76d40cc0879df32bc342d	-0.369403779	0.387934913	k__Bacteria; p__Firmicutes; c__Clostridia; o__Clostridiales
fda53e1a26f7489a683cfe01228fca8f	-1.315664232	0.397583058	k__Bacteria; p__Firmicutes; c__Clostridia; o__Clostridiales; f__Lachnospiraceae; g__Clostridium; s__lavalense
8f98fb8693ed59c21399d83ce2d10724	3.21970667	0.421861579	k__Bacteria; p__Verrucomicrobia; c__Verrucomicrobiae; o__Verrucomicrobiales; f__Verrucomicrobiaceae; g__Akkermansia; s__muciniphila
cadafbd17c8a30e17ae4b16c1caa1b69	-1.509799897	0.426450422	k__Bacteria; p__Firmicutes; c__Clostridia; o__Clostridiales; f__[Tissierellaceae]; g__WAL_1855D; s__
568a63615bbf7c37fe5a9876a2680db5	-0.756976545	0.426753511	k__Bacteria; p__Proteobacteria; c__Deltaproteobacteria; o__Desulfovibrionales; f__Desulfovibrionaceae; g__Desulfovibrio; s__
543a0a3f73c2932bab06a3c737bfef5b	0.901225388	0.434350064	k__Bacteria; p__Firmicutes; c__Clostridia; o__Clostridiales; f__Lachnospiraceae; g__Lachnospira; s__
dfd770d48651635c39bdafe13f62e75a	0.787557662	0.440736821	k__Bacteria; p__Proteobacteria; c__Deltaproteobacteria; o__Desulfovibrionales; f__Desulfovibrionaceae; g__Desulfovibrio; s__
c4f4806bb9adcbc03460e779d8f42b48	-0.751905381	0.441155245	k__Bacteria; p__Bacteroidetes; c__Bacteroidia; o__Bacteroidales; f__[Barnesiellaceae]; g__; s__
c88e37c00a5abc483dada3afc5da38a3	1.396501363	0.462479999	k__Bacteria; p__Firmicutes; c__Clostridia; o__Clostridiales; f__Ruminococcaceae
f0111bac5234f907953885881c7e241c	1.723555506	0.467334022	k__Bacteria; p__Bacteroidetes; c__Bacteroidia; o__Bacteroidales; f__Bacteroidaceae; g__Bacteroides; s__ovatus
33d591dd6da939cd5ffa983ad2b49280	-0.629682481	0.476404896	k__Bacteria; p__Bacteroidetes; c__Bacteroidia; o__Bacteroidales; f__Prevotellaceae; g__Prevotella; s__
bfd6fb8bbe55a7f01443710620765c22	-1.408521116	0.477373292	k__Bacteria; p__Firmicutes; c__Clostridia; o__Clostridiales; f__Ruminococcaceae; g__Gemmiger; s__formicilis
01fdc2c336a8193e8aa4de23d7c09e33	-2.188377797	0.487606158	k__Bacteria; p__Firmicutes; c__Clostridia; o__Clostridiales; f__Lachnospiraceae
351c1c41eb96b3ed2975bfe7f01eb9d7	3.859515459	0.513851812	k__Bacteria; p__Bacteroidetes; c__Bacteroidia; o__Bacteroidales; f__Prevotellaceae; g__Prevotella; s__copri
e27680d4009f98f30248d823bc17fb8e	1.400644362	0.51734667	k__Bacteria; p__Proteobacteria; c__Gammaproteobacteria; o__Pasteurellales; f__Pasteurellaceae; g__Haemophilus; s__parainfluenzae
7b88642b16527112c71d6cf714323e5d	-0.06478399	0.520991077	k__Bacteria; p__Firmicutes; c__Clostridia; o__Clostridiales; f__Lachnospiraceae; g__Coprococcus; s__
492f380604907b04349e50a01a091656	-0.294012248	0.523654809	k__Bacteria; p__Bacteroidetes; c__Bacteroidia; o__Bacteroidales; f__[Barnesiellaceae]; g__; s__
22c8e6e55e7d3f64fc2fdf7b5cc181a2	-2.779318273	0.526101699	k__Bacteria; p__Firmicutes; c__Clostridia; o__Clostridiales; f__Lachnospiraceae; g__Clostridium; s__aldenense

c1310e0b3b6813e4558e82286ab4f8d7	-1.295964658	0.533104112	k__Bacteria; p__Firmicutes; c__Clostridia; o__Clostridiales; f__Lachnospiraceae; g__[Ruminococcus]; s__
13674ec8c190b0add808fa9c2e1ca466	1.918653548	0.536609759	k__Bacteria; p__Firmicutes; c__Clostridia; o__Clostridiales; f__Veillonellaceae; g__Acidaminococcus; s__
bcc7da211cfd8c6f2b18577d26787e5d	-1.526750505	0.536929478	k__Bacteria; p__Bacteroidetes; c__Bacteroidia; o__Bacteroidales; f__Porphyromonadaceae; g__Parabacteroides; s__distasonis
f95cab37fba4160de15015f4d520839f	2.736182452	0.548173716	k__Bacteria; p__Firmicutes; c__Erysipelotrichi; o__Erysipelotrichales; f__Erysipelotrichaceae; g__; s__
cd287f4187715ce042afa01534b95a9b	0.865410865	0.56843399	k__Bacteria; p__Firmicutes; c__Clostridia; o__Clostridiales; f__Lachnospiraceae; g__Coprococcus; s__
d6960828e54e3b0059ec5cdf24b16d2f	2.02813834	0.572249522	k__Bacteria; p__Firmicutes; c__Clostridia; o__Clostridiales; f__Veillonellaceae; g__Dialister; s__
01ae78da1e1ad9622c03153c190425ef	-2.749005735	0.575834086	k__Bacteria; p__Firmicutes; c__Clostridia; o__Clostridiales; f__Lachnospiraceae
9244dfd9a167be2a99a8b77f6297dbf6	-1.989889085	0.577007224	k__Bacteria; p__Firmicutes; c__Clostridia; o__Clostridiales; f__Peptostreptococcaceae; g__[Clostridium]; s__bifermentans
1d5c469bc3a0da4471c67e502fa56e36	-2.504919469	0.579522719	k__Bacteria; p__Firmicutes; c__Clostridia; o__Clostridiales; f__Eubacteriaceae; g__Pseudoramibacter_Eubacterium; s__
7534fb513a4b404419edc4e91920af3f	1.13755852	0.581139972	k__Bacteria; p__Bacteroidetes; c__Bacteroidia; o__Bacteroidales; f__Rikenellaceae; g__Alistipes; s__onderdonkii
0a4009eb300a0f2671383587d6d8401e	-2.964419305	0.581249168	k__Bacteria; p__Firmicutes; c__Bacilli; o__Lactobacillales; f__Streptococcaceae; g__Streptococcus
677929eac52be081cdae08a0b9c70eb2	4.084889531	0.591388991	k__Bacteria; p__Proteobacteria; c__Gammaproteobacteria; o__Enterobacteriales; f__Enterobacteriaceae
f9ff9d5eb1c59d26a9689d1b617eba2b	-2.581666887	0.593456437	k__Bacteria; p__Firmicutes; c__Bacilli; o__Lactobacillales; f__Streptococcaceae; g__Streptococcus; s__anginosus
10ee6e361d2a11328a3814ddc8c3a2c5	2.667066396	0.61579453	k__Bacteria; p__Bacteroidetes; c__Bacteroidia; o__Bacteroidales; f__Prevotellaceae; g__Prevotella; s__stercorea
ec4075339e16f5cd45fd5a7955596899	3.415056937	0.619862845	k__Bacteria; p__Bacteroidetes; c__Bacteroidia; o__Bacteroidales; f__Bacteroidaceae; g__Bacteroides; s__caccae
a78fa572a60226d8150bfb0fc0fad652	-2.659173906	0.645182719	k__Bacteria; p__Actinobacteria; c__Actinobacteria; o__Actinomycetales; f__Actinomycetaceae; g__Actinomyces; s__
49f1abaf33a12ddf88f3cccd70883c68	-1.049992025	0.646616807	k__Bacteria; p__Firmicutes; c__Clostridia; o__Clostridiales; f__Ruminococcaceae
52d170b1be2ba3e00962d97f39f6670d	-2.132851541	0.649591198	k__Bacteria; p__Bacteroidetes; c__Bacteroidia; o__Bacteroidales; f__Porphyromonadaceae; g__Parabacteroides; s__distasonis
5765b6cad3f3850022d8c5093cd84879	0.879402459	0.652335098	k__Bacteria; p__Bacteroidetes; c__Bacteroidia; o__Bacteroidales; f__[Paraprevotellaceae]; g__Paraprevotella; s__
119295098ad0c31b01ddf55773a9167c	-1.314135968	0.654624274	k__Bacteria; p__Firmicutes; c__Erysipelotrichi; o__Erysipelotrichales; f__Erysipelotrichaceae; g__; s__
f30ac5063ec37bc24a902343326db800	-0.475943982	0.660848489	k__Bacteria; p__Firmicutes; c__Clostridia; o__Clostridiales; f__Lachnospiraceae; g__; s__
81a68ce839334e658fe99db4f3f0f012	-1.066135347	0.674326589	k__Bacteria; p__Proteobacteria; c__Betaproteobacteria; o__Burkholderiales; f__Alcaligenaceae; g__Sutterella; s__
7d285be20e3ad3812eb21be379357ef1	3.095740766	0.680129101	k__Bacteria; p__Firmicutes; c__Clostridia; o__Clostridiales; f__Lachnospiraceae; g__Roseburia
25d727166a36df8d2f6a915a945bf5ac	4.114912391	0.681354096	k__Bacteria; p__Bacteroidetes; c__Bacteroidia; o__Bacteroidales; f__Bacteroidaceae; g__Bacteroides
bf0ed76ac8b022b5896f58f3085b0d5e	0.114605487	0.682617595	k__Bacteria; p__Firmicutes; c__Clostridia; o__Clostridiales; f__Ruminococcaceae; g__Oscillospira; s__
fe8cb22c3ddf1ab8b026a68c1ded28a1	-1.513060987	0.683856597	k__Bacteria; p__Firmicutes; c__Clostridia; o__Clostridiales; f__Lachnospiraceae
afd87e82de329a1ed75b98b5b606843c	1.14785105	0.696493318	k__Bacteria; p__Firmicutes; c__Clostridia; o__Clostridiales; f__Lachnospiraceae; g__Dorea; s__formicigenerans
23aac0307eb1233906a488304e122cb2	0.481286109	0.698492756	k__Bacteria; p__Firmicutes; c__Clostridia; o__Clostridiales; f__Lachnospiraceae

ff2bd29ff42e4dc25a31714e0b6c2dca	-2.191800058	0.69928961	k__Bacteria; p__Firmicutes; c__Bacilli; o__Lactobacillales; f__Lactobacillaceae; g__Lactobacillus
cb63ff338fde1171b1e985e8211b1929	1.508653939	0.714001407	k__Bacteria; p__Firmicutes; c__Clostridia; o__Clostridiales; f__Ruminococcaceae; g__Oscillospira; s__
b8c804e73bd7b96d1407eb34ab4f27d2	-0.948616445	0.718649497	k__Bacteria; p__Firmicutes; c__Clostridia; o__Clostridiales
d38ece5f6399b4f8a676e21888b813af	2.929077447	0.727673849	k__Bacteria; p__Bacteroidetes; c__Bacteroidia; o__Bacteroidales; f__Bacteroidaceae; g__Bacteroides; s__plebeius
85ae543a3d043a13f3df732cb614682f	1.16447264	0.735964467	k__Bacteria; p__Bacteroidetes; c__Bacteroidia; o__Bacteroidales; f__[Paraprevotellaceae]; g__Paraprevotella; s__
cd2422c66d54d6e8ad1ba9bd6761b2f8	-0.427929103	0.740175297	k__Bacteria; p__Firmicutes; c__Clostridia; o__Clostridiales; f__Lachnospiraceae; g__Clostridium; s__hathewayi
a4e94137d3eea12b54dec05caedafe47	1.937144697	0.742263248	k__Bacteria; p__Firmicutes; c__Clostridia; o__Clostridiales; f__Lachnospiraceae
e5413f67faa6b8c0c3e63e48836c0b42	2.372905553	0.757091989	k__Bacteria; p__Firmicutes; c__Clostridia; o__Clostridiales; f__Veillonellaceae; g__Phascolarctobacterium; s__
e553b9a0bb32467c71c89a4e97e55792	1.844422043	0.773074707	k__Bacteria; p__Firmicutes; c__Clostridia; o__Clostridiales; f__Ruminococcaceae; g__Faecalibacterium; s__prausnitzii
ece50a62168f85fc61385d8adb4c6494	1.610891044	0.778789213	k__Bacteria; p__Proteobacteria; c__Deltaproteobacteria; o__Desulfovibrionales; f__Desulfovibrionaceae; g__Bilophila; s__
8aa94826eb4f90512d52fc7b796354c6	0.626543105	0.781141391	k__Bacteria; p__Firmicutes; c__Clostridia; o__Clostridiales; f__Ruminococcaceae; g__Ruminococcus; s__
877cbd485e8e00858a551153bb630d8e	-1.227985799	0.784520974	k__Bacteria; p__Firmicutes; c__Clostridia; o__Clostridiales; f__Ruminococcaceae; g__Oscillospira; s__
90a05d597112b554e4480a8eaae4e0aa	2.074935496	0.790290197	k__Bacteria; p__Firmicutes; c__Clostridia; o__Clostridiales; f__Lachnospiraceae
caab3b52768392d41ad00f268e7486a2	-1.999209344	0.791535904	k__Bacteria; p__Proteobacteria; c__Deltaproteobacteria; o__Desulfovibrionales; f__Desulfovibrionaceae; g__Desulfovibrio; s__
b39c338f5e964b6cb87e07f10badc6c4	-1.722663343	0.802498838	k__Bacteria; p__Firmicutes; c__Bacilli; o__Lactobacillales; f__Streptococcaceae; g__Lactococcus; s__
3baabf8cc3e85c0f8b1d2790db20f838	-2.943508088	0.805177023	k__Bacteria; p__Actinobacteria; c__Actinobacteria; o__Actinomycetales; f__Micrococcaceae; g__Rothia; s__mucilaginosa
c950846150e92b59f7b0629afe0c0060	-0.554270923	0.816656997	k__Bacteria; p__Firmicutes; c__Clostridia; o__Clostridiales; f__Peptostreptococcaceae
8347bd34436f72573fcde614b95d4702	2.364711345	0.818801006	k__Bacteria; p__Actinobacteria; c__Actinobacteria; o__Bifidobacteriales; f__Bifidobacteriaceae; g__Bifidobacterium; s__
2acaf275cc5b6e89e0f9f52c9197bbf7	-0.425675332	0.819732984	k__Bacteria; p__Firmicutes; c__Erysipelotrichi; o__Erysipelotrichales; f__Erysipelotrichaceae; g__[Eubacterium]; s__biforme
d624f3cc54840847422f1cf7b6ad4a1d	0.293840707	0.838398387	k__Bacteria; p__Proteobacteria; c__Betaproteobacteria; o__Burkholderiales; f__Alcaligenaceae; g__Sutterella; s__
97e403cfc9503f262fb9e25075a23129	-0.478521526	0.85525467	k__Bacteria; p__Firmicutes; c__Clostridia; o__Clostridiales; f__Ruminococcaceae; g__Gemmiger; s__formicilis
01c354063f81063d367ced12e5ce1528	-0.890359342	0.86337774	k__Bacteria; p__Bacteroidetes; c__Bacteroidia; o__Bacteroidales; f__Bacteroidaceae; g__Bacteroides; s__ovatus
228ae07feb0040900651f7580168bd27	0.034719527	0.869006713	k__Bacteria; p__Firmicutes; c__Erysipelotrichi; o__Erysipelotrichales; f__Erysipelotrichaceae; g__Clostridium; s__ramosum
142a9c61213ee7480a1c7b0eee5fc2c7	-1.109602391	0.874272039	k__Bacteria; p__Firmicutes; c__Erysipelotrichi; o__Erysipelotrichales; f__Erysipelotrichaceae; g__[Eubacterium]; s__dolichum
0bc0309dd21fa1eba946576bc9ef55d8	0.035090506	0.883000573	k__Bacteria; p__Firmicutes; c__Bacilli; o__Lactobacillales; f__Lactobacillaceae; g__Lactobacillus; s__ruminis
719d048052a9dc2af9658615fc430339	-2.642074525	0.895411333	k__Bacteria; p__Firmicutes; c__Bacilli; o__Lactobacillales; f__Enterococcaceae; g__Enterococcus
651b456fad22829867a61aff2a233caa	-0.715687692	0.90042602	k__Bacteria; p__Firmicutes; c__Bacilli; o__Lactobacillales; f__Leuconostocaceae

73bf8d1a5983e34a0cb84e3cae 127815	0.516409 457	0.900606682	k__Bacteria; p__Firmicutes; c__Bacilli; o__Lactobacillales; f__Streptococcaceae; g__Streptococcus; s__
9d21d7d00d7e938a7e1c374a4f 4bb833	- 1.469545 305	0.906168153	k__Bacteria; p__Firmicutes; c__Clostridia; o__Clostridiales; f__Lachnospiraceae
6bb6aad559c5eaeb22b2eef787 4ef038	- 1.201208 532	0.906663587	k__Bacteria; p__Actinobacteria; c__Coriobacteriia; o__Coriobacteriales; f__Coriobacteriaceae; g__Eggerthella; s__lenta
534ad0c60e501c4553d4cbae8 0ccd5ab	- 2.210577 428	0.907795539	k__Bacteria; p__Bacteroidetes; c__Bacteroidia; o__Bacteroidales; f__Porphyromonadaceae; g__Parabacteroides; s__distasonis
e59405a47acbc248ce6139536 6159d8d	1.923616 35	0.917457362	k__Bacteria; p__Firmicutes; c__Clostridia; o__Clostridiales; f__Lachnospiraceae; g__[Ruminococcus]; s__torques
4ff2e9004a1262952381951a17 3485af	0.876754 821	0.921581259	k__Bacteria; p__Firmicutes; c__Clostridia; o__Clostridiales; f__Lachnospiraceae; g__Dorea; s__
d12873ac6a9f7f5c1e489de5ee 122167	- 1.671271 741	0.92522957	k__Bacteria; p__Firmicutes; c__Clostridia; o__Clostridiales; f__Lachnospiraceae; g__Clostridium
a1a3200b76bcd6000a0914892 d370b6e	0.077744 067	0.926126768	k__Bacteria; p__Firmicutes; c__Bacilli; o__Lactobacillales; f__Enterococcaceae; g__Enterococcus
000e38f477632fa6299901badf 2e0f06	- 1.139056 623	0.936091294	k__Bacteria; p__Firmicutes; c__Bacilli; o__Lactobacillales; f__Streptococcaceae; g__Streptococcus
5d6ee23084c6b9c96deb9a832 95abc8a	- 2.050208 032	0.948188146	k__Bacteria; p__Firmicutes; c__Bacilli; o__Lactobacillales; f__Streptococcaceae; g__Streptococcus
0088553fbbbf2fbc8918ad2245 57d65c	1.368941 129	0.995322248	k__Bacteria; p__Proteobacteria; c__Betaproteobacteria; o__Burkholderiales; f__Alcaligenaceae; g__Sutterella; s__
03feb113c217dbf05d6fa6974b 302490	- 3.022722 661	1.008952161	k__Bacteria; p__Bacteroidetes; c__Bacteroidia; o__Bacteroidales; f__Porphyromonadaceae; g__Parabacteroides; s__distasonis
aa9640375ef8e0ef404343bd2e d52edd	- 1.737189 71	1.026278829	k__Bacteria; p__Firmicutes; c__Clostridia; o__Clostridiales; f__Peptostreptococcaceae; g__[Clostridium]; s__sordellii
69e611251f4d8582e312afa573 7f033e	2.091318 31	1.035542553	k__Bacteria; p__Actinobacteria; c__Actinobacteria; o__Bifidobacteriales; f__Bifidobacteriaceae; g__Bifidobacterium
dfba68ef0fd0e712608eb2a007 8013a7	1.813664 019	1.037544032	k__Bacteria; p__Actinobacteria; c__Coriobacteriia; o__Coriobacteriales; f__Coriobacteriaceae; g__Collinsella; s__aerofaciens
e083e2f58987c5f8db5d4dd16d dde91f	- 2.922037 542	1.05568931	k__Bacteria; p__Firmicutes; c__Erysipelotrichi; o__Erysipelotrichales; f__Erysipelotrichaceae; g__Bulleidia; s__moorei
7b223719a0af567e7ea99f06f7 ea1068	0.075192 511	1.065258955	k__Bacteria; p__Actinobacteria; c__Actinobacteria; o__Bifidobacteriales; f__Bifidobacteriaceae; g__Bifidobacterium; s__bifidum
12391d25fe187094cfd00f1b3d 5b205a	- 1.181183 278	1.112244544	k__Bacteria; p__Firmicutes; c__Clostridia; o__Clostridiales; f__Veillonellaceae; g__Acidaminococcus; s__
2b4a79f2b8db74f757ce83fc8c de5b5d	1.012083 113	1.112809939	k__Bacteria; p__Bacteroidetes; c__Bacteroidia; o__Bacteroidales; f__Prevotellaceae; g__Prevotella; s__stercorea
19a6175fceab96a4ababe9a61f 175cdf	- 1.099648 416	1.135789206	k__Bacteria; p__Firmicutes; c__Clostridia; o__Clostridiales; f__Lachnospiraceae
c2bbed9f2d4200fb3f814c7741 5026f9	- 3.132277 906	1.139723984	k__Bacteria; p__Firmicutes; c__Bacilli; o__Lactobacillales; f__Carnobacteriaceae
8db356aa7494262040e1b0f4c 88f1a8d	- 3.148400 724	1.155712483	k__Bacteria; p__Firmicutes; c__Clostridia; o__Clostridiales
0c409483330f4e4cd40a713f84 4f6661	- 2.425989 091	1.176866008	k__Bacteria; p__Firmicutes; c__Clostridia; o__Clostridiales; f__Ruminococcaceae; g__Oscillospira; s__
406acdfe0cff73b824bd07ddf9 036e04	- 2.276272 237	1.184966823	k__Bacteria; p__Proteobacteria; c__Gammaproteobacteria; o__Enterobacteriales; f__Enterobacteriaceae
136c7191e0643043625a12681 29bac61	- 0.731588 781	1.201017834	k__Bacteria; p__Fusobacteria; c__Fusobacteriia; o__Fusobacteriales; f__Fusobacteriaceae; g__Fusobacterium; s__
bd2ebc70501f7d867c204f94c4 e483da	1.778025 806	1.20378308	k__Bacteria; p__Firmicutes; c__Bacilli; o__Lactobacillales; f__Streptococcaceae; g__Streptococcus; s__
b65eb19257f7a2bedb5a1c4b42 aeb396	2.811287 403	1.212728879	k__Bacteria; p__Bacteroidetes; c__Bacteroidia; o__Bacteroidales; f__Bacteroidaceae; g__Bacteroides; s__

3df3f3507535f086e675753cc4e7e7b5	3.43336016	1.224504163	k__Bacteria; p__Firmicutes; c__Clostridia; o__Clostridiales; f__Veillonellaceae; g__Megamonas; s__
2c2018d4ecccfa3cb27b99a04c9222b1	1.38073808	1.25310808	k__Bacteria; p__Bacteroidetes; c__Bacteroidia; o__Bacteroidales; f__Bacteroidaceae; g__Bacteroides; s__ovatus
68baf6c0a24d8a4eb53ce1405fedde5f	-2.698227346	1.263006014	k__Bacteria; p__Firmicutes; c__Clostridia; o__Clostridiales; f__Lachnospiraceae
7fb5cef2a6415dfc9342723dfadd8515	0.155946315	1.281729119	k__Bacteria; p__Bacteroidetes; c__Bacteroidia; o__Bacteroidales; f__Bacteroidaceae; g__Bacteroides
4b347c4343a20c5aa17d05f1bcf3a8e9	-1.840284764	1.33160798	k__Bacteria; p__Firmicutes; c__Clostridia; o__Clostridiales; f__; g__; s__
90eca2d0a5abab90568906f8acb13efa	-1.498103082	1.333683421	k__Bacteria; p__Firmicutes; c__Clostridia; o__Clostridiales; f__Lachnospiraceae; g__[Ruminococcus]; s__
c88b37a81d4bafa4f4c8c87bf94b38a5	-1.657486379	1.336427739	k__Bacteria; p__Bacteroidetes; c__Bacteroidia; o__Bacteroidales; f__Bacteroidaceae
4c06188bc9c7b91eec9e1fe25da00706	-2.881085813	1.345111264	k__Bacteria; p__Actinobacteria; c__Actinobacteria; o__Actinomycetales; f__Actinomycetaceae; g__Actinomyces; s__
df72998479c8b1228c5c3943f047680b	-2.773231923	1.357466554	k__Bacteria; p__Actinobacteria; c__Actinobacteria; o__Actinomycetales; f__Actinomycetaceae; g__Actinomyces; s__
9496d87b94d90dff068f0716603930bd	2.583885372	1.358854895	k__Bacteria; p__Bacteroidetes; c__Bacteroidia; o__Bacteroidales; f__Bacteroidaceae; g__Bacteroides; s__fragilis
9f4bbb193b0d460b76e81c1423dc9860	-0.16613549	1.363044744	k__Bacteria; p__Bacteroidetes; c__Bacteroidia; o__Bacteroidales; f__Bacteroidaceae; g__Bacteroides; s__caccae
608e6548b1b4cbb6176c8fce090991a5	-1.941044271	1.372882044	k__Bacteria; p__Actinobacteria; c__Actinobacteria; o__Actinomycetales; f__Actinomycetaceae; g__Actinomyces; s__
8133592a0dd3d6f104c1299d656af362	0.869829953	1.375172933	k__Bacteria; p__Firmicutes; c__Clostridia; o__Clostridiales; f__Clostridiaceae; g__Clostridium; s__celatum
6a0bbfe5241c80e0f4e91b06fe4715c7	1.014496386	1.380656128	k__Bacteria; p__Firmicutes; c__Clostridia; o__Clostridiales; f__Clostridiaceae; g__Clostridium; s__celatum
2ea35c2aa510ab4f9fbc93c8e65bdd51	3.320854061	1.38127849	k__Bacteria; p__Proteobacteria; c__Gammaproteobacteria; o__Aeromonadales; f__Succinivibrionaceae; g__Succinivibrio; s__
5d564d5db4ddb67c61b7fdfbffbe15d3	-3.14757961	1.384252598	k__Bacteria; p__Actinobacteria; c__Actinobacteria; o__Bifidobacteriales; f__Bifidobacteriaceae; g__Bifidobacterium; s__
392aa3c45e128c7432f91b3de84541f6	0.578800977	1.392325868	k__Bacteria; p__Firmicutes; c__Clostridia; o__Clostridiales; f__Lachnospiraceae; g__Clostridium; s__citroniae
0c87c8c664745907a533973b395c37bb	-2.487310827	1.395899018	k__Bacteria; p__Bacteroidetes; c__Bacteroidia; o__Bacteroidales; f__Porphyromonadaceae; g__Parabacteroides; s__
974f6d026e2fa28ed37bcc0308ed56cf	2.713428736	1.40563639	k__Bacteria; p__Bacteroidetes; c__Bacteroidia; o__Bacteroidales; f__Bacteroidaceae; g__Bacteroides; s__
6a6fcf8f9b8bb1ab9e5f8456ee7fb109	1.731788814	1.408738201	k__Bacteria; p__Firmicutes; c__Clostridia; o__Clostridiales; f__Lachnospiraceae; g__[Ruminococcus]; s__
00565f9981f176f2eae21aff895a72ff	-0.16134566	1.422691961	k__Bacteria; p__Firmicutes; c__Clostridia; o__Clostridiales; f__Lachnospiraceae; g__[Ruminococcus]; s__
37a90abf181b684611b3eabfd5dfa715	-2.241332948	1.43370161	k__Bacteria; p__Proteobacteria; c__Gammaproteobacteria; o__Enterobacteriales; f__Enterobacteriaceae; g__Proteus; s__
1e3e4d8ace0f5acef651d3c5038d743f	0.3588987	1.44487614	k__Bacteria; p__Firmicutes; c__Clostridia; o__Clostridiales; f__Clostridiaceae; g__Clostridium; s__perfringens
a73a945152632348be2f01c58d846887	-3.11339277	1.447150414	k__Bacteria; p__Firmicutes; c__Clostridia; o__Clostridiales; f__Lachnospiraceae
23169183de10f35b5239c7f337ace0c0	-2.96122688	1.462340837	k__Bacteria; p__Bacteroidetes; c__Bacteroidia; o__Bacteroidales; f__Porphyromonadaceae; g__Parabacteroides
0fe8fe3f1c76fe3c1a9090a9b87cff51	-2.235661924	1.471542006	k__Bacteria; p__Bacteroidetes; c__Bacteroidia; o__Bacteroidales; f__[Odoribacteraceae]; g__Odoribacter; s__
5608c3e6c9de9ceb79610e7786bd0ac4	-0.744766652	1.530410369	k__Bacteria; p__Firmicutes; c__Clostridia; o__Clostridiales; f__Veillonellaceae; g__Veillonella; s__dispar

e0901a2b53c0165a5a09e451794c8839	-2.43811363	1.54464825	k__Bacteria; p__Actinobacteria; c__Coriobacteriia; o__Coriobacteriales; f__Coriobacteriaceae; g__Collinsella; s__
45a68a9eee3cf83e27f4ea309d57ffc3	-1.953667104	1.575436016	k__Bacteria; p__Firmicutes; c__Bacilli; o__Lactobacillales; f__Lactobacillaceae; g__Lactobacillus; s__zeae
85b4d7d480ef06ef2844b93115d87480	1.141493857	1.598621806	k__Bacteria; p__Firmicutes; c__Clostridia; o__Clostridiales; f__Veillonellaceae; g__Megasphaera; s__
a26864c466353615ac05a60b7f5a199c	-2.653565824	1.641861667	k__Bacteria; p__Firmicutes; c__Erysipelotrichi; o__Erysipelotrichales; f__Erysipelotrichaceae; g__[Eubacterium]; s__dolichum
06296ef8e5b973cb2ac066c552af976a	-0.545949876	1.661553492	k__Bacteria; p__Firmicutes; c__Clostridia; o__Clostridiales; f__Peptostreptococcaceae
7533ad859560f7a061e4bcc4e60e25ec	1.21602875	1.666429033	k__Bacteria; p__Firmicutes; c__Clostridia; o__Clostridiales; f__Lachnospiraceae
4f0327d92b133d32fecd7992814c7398	-0.659824311	1.69288822	k__Bacteria; p__Firmicutes; c__Clostridia; o__Clostridiales; f__Lachnospiraceae; g__Clostridium; s__hathewayi
4cac188f212a24bea2a1768192189c77	-1.394487798	1.70240139	k__Bacteria; p__Firmicutes; c__Bacilli; o__Lactobacillales; f__Streptococcaceae; g__Streptococcus; s__luteciae
502d55e4bd13f0aaf1d4a9123cb25580	-1.7982077	1.708941361	k__Bacteria; p__Firmicutes; c__Erysipelotrichi; o__Erysipelotrichales; f__Erysipelotrichaceae; g__Coprobacillus; s__cateniformis
d1fe724ddc2611a0d22789516d940a6d	-3.065813958	1.709432562	k__Bacteria; p__Firmicutes; c__Erysipelotrichi; o__Erysipelotrichales; f__Erysipelotrichaceae; g__[Eubacterium]; s__dolichum
a5189f77a2cfeab3bc1602ff5c8ac3e9	-1.818782747	1.744919708	k__Bacteria; p__Firmicutes; c__Bacilli; o__Lactobacillales; f__Streptococcaceae; g__Streptococcus
c0620081a043535f0714ef4e4fe1aef8	1.782454074	1.750231614	k__Bacteria; p__Firmicutes; c__Clostridia; o__Clostridiales; f__Veillonellaceae; g__Megasphaera; s__
bfe54af4c9180d37a0d76f6dafe79a5a	-1.474603116	1.752169182	k__Bacteria; p__Proteobacteria; c__Gammaproteobacteria; o__Enterobacteriales; f__Enterobacteriaceae
daff4ecf4d60b34bb90f08bedf2a9b34	-0.05078119	1.75270777	k__Bacteria; p__Bacteroidetes; c__Bacteroidia; o__Bacteroidales; f__[Paraprevotellaceae]; g__Paraprevotella; s__
2731573ea86a7bedc3f7c2ee9e510e40	-3.016694486	1.872557928	k__Bacteria; p__Firmicutes; c__Clostridia; o__Clostridiales; f__Lachnospiraceae; g__Blautia; s__producta
5173dbed1ba24aca7d164bf37813de35	0.588887036	1.88715147	k__Bacteria; p__Firmicutes; c__Clostridia; o__Clostridiales; f__Veillonellaceae; g__Dialister; s__
8f385d71fcd6fc1e94d77678f279c04a	-0.000189721	1.889101138	k__Bacteria; p__Firmicutes; c__Clostridia; o__Clostridiales; f__Ruminococcaceae; g__Faecalibacterium; s__prausnitzii
2f7469e5218c41b52377f7a2423038d6	-1.687315404	1.894243767	k__Bacteria; p__Firmicutes; c__Clostridia; o__Clostridiales; f__Lachnospiraceae; g__Blautia; s__
6627f143de487e3c431333901080439d	0.206187547	1.904058029	k__Bacteria; p__Fusobacteria; c__Fusobacteriia; o__Fusobacteriales; f__Fusobacteriaceae
f7a76964e122c049fa28708d3880b576	-2.49571079	1.939533403	k__Bacteria; p__Bacteroidetes; c__Bacteroidia; o__Bacteroidales; f__Bacteroidaceae; g__Bacteroides; s__uniformis
17933bd4ac9b7983d192c4014a40dbff	-2.040139615	1.939535191	k__Bacteria; p__Bacteroidetes; c__Bacteroidia; o__Bacteroidales; f__[Paraprevotellaceae]; g__Paraprevotella; s__
fac622734a092cd5e3113b2f103ccde7	-2.626904905	1.958660831	k__Bacteria; p__Firmicutes; c__Clostridia; o__Clostridiales; f__Lachnospiraceae; g__Dorea; s__
2f27bcf9a60804b6b1fcdf74122c4fae	-0.232395351	2.014339616	k__Bacteria; p__Firmicutes; c__Clostridia; o__Clostridiales; f__Lachnospiraceae; g__Dorea; s__
cc2d96099f530b503371e5ddca8c0a58	1.418508351	2.034132709	k__Bacteria; p__Firmicutes; c__Clostridia; o__Clostridiales; f__Lachnospiraceae; g__[Ruminococcus]; s__gnavus
9154fa6a1f8d4e5c562994121242f5b5	-1.516562879	2.065255036	k__Bacteria; p__Firmicutes; c__Clostridia; o__Clostridiales; f__Lachnospiraceae
464a52deb6e8c4e2b57d980a8d18f04c	2.357218802	2.084428539	k__Bacteria; p__Firmicutes; c__Clostridia; o__Clostridiales; f__Peptostreptococcaceae; g__; s__
a4bc8ec02405aa22556bf3c19686cf67	-1.900052011	2.089817872	k__Bacteria; p__Bacteroidetes; c__Bacteroidia; o__Bacteroidales; f__Bacteroidaceae; g__Bacteroides; s__ovatus

4f363011ef8cbd14bc5502b6d631d171	-3.026207387	2.104958405	k__Bacteria; p__Firmicutes; c__Bacilli; o__Lactobacillales; f__Lactobacillaceae; g__Lactobacillus; s__mucosae
2ba3d32f68a0a0eed0b77fbb16f8e49f	-1.291839063	2.121840229	k__Bacteria; p__Bacteroidetes; c__Bacteroidia; o__Bacteroidales; f__[Paraprevotellaceae]; g__Paraprevotella; s__
5b4f8b625d8fbb1268863be7dbc4db5d	-2.260561406	2.148998549	k__Bacteria; p__Firmicutes; c__Clostridia; o__Clostridiales; f__Veillonellaceae; g__Veillonella; s__dispar
7b0976f8022adcc58c0770e3ac755cc9	1.388742745	2.156430831	k__Bacteria; p__Fusobacteria; c__Fusobacteriia; o__Fusobacteriales; f__Fusobacteriaceae
2806d865c2799de96eee6442e234d8ea	-1.185961663	2.165842166	k__Bacteria; p__Firmicutes; c__Clostridia; o__Clostridiales; f__Lachnospiraceae
cda4e6f933bb3108ea3e92f9db411c00	-2.581194341	2.207715621	k__Bacteria; p__Firmicutes; c__Bacilli; o__Gemellales; f__Gemellaceae
8dfe50db912cbbd6fd80a14415e2d530	-1.61084789	2.382293632	k__Bacteria; p__Proteobacteria; c__Deltaproteobacteria; o__Desulfovibrionales; f__Desulfovibrionaceae; g__Bilophila; s__
0f5f910af5780b36149bc45258033686	-1.991145551	2.468031933	k__Bacteria; p__Bacteroidetes; c__Bacteroidia; o__Bacteroidales; f__Prevotellaceae; g__Prevotella; s__stercorea
c505404abf2952958e1f438217081417	0.446214736	2.476891806	k__Bacteria; p__Verrucomicrobia; c__Verrucomicrobiae; o__Verrucomicrobiales; f__Verrucomicrobiaceae; g__Akkermansia; s__muciniphila
3ab752181b720a897cf1adc0c7670bf9	-0.953274667	2.54428499	k__Bacteria; p__Firmicutes; c__Clostridia; o__Clostridiales; f__Lachnospiraceae; g__Blautia; s__
501bafa20cbe37ff2cb4886f5e7fc07d	-1.379410207	2.554472616	k__Bacteria; p__Fusobacteria; c__Fusobacteriia; o__Fusobacteriales; f__Fusobacteriaceae
e1d2a9fa4aa3f8e81067cf362e0cd257	-0.12133044	2.604734709	k__Bacteria; p__Firmicutes; c__Bacilli; o__Turicibacterales; f__Turicibacteraceae; g__Turicibacter; s__
bb5095d1e0f85abd923860c199d75c48	-1.08033365	2.618965556	k__Bacteria; p__Firmicutes; c__Clostridia; o__Clostridiales; f__Clostridiaceae; g__SMB53; s__
8c4d5ca7271a3b4402f17b1e04cbcd1f	-0.025065839	2.622009446	k__Bacteria; p__Firmicutes; c__Clostridia; o__Clostridiales; f__Clostridiaceae; g__Clostridium; s__hiranonis
d88daea65b6cdeaab41de5bd59e92c60	-2.92220062	2.69615381	k__Bacteria; p__Firmicutes; c__Clostridia; o__Clostridiales; f__Lachnospiraceae; g__Blautia; s__producta
1dcfecb2b27d824c4260fcfe06c50f12	-2.622573793	2.711539438	k__Bacteria; p__Firmicutes; c__Clostridia; o__Clostridiales; f__Lachnospiraceae; g__Blautia; s__producta
158782cefa6e28406a2a0aeb75220f0a	-2.821734845	2.846895625	k__Bacteria; p__Bacteroidetes; c__Bacteroidia; o__Bacteroidales; f__[Barnesiellaceae]; g__; s__
5367c25a87f2b25819f9f7803785d573	-1.732565343	2.893090536	k__Bacteria; p__Actinobacteria; c__Coriobacteriia; o__Coriobacteriales; f__Coriobacteriaceae; g__Collinsella; s__stercoris
d353248c8a7d31bc9f8378a320b77924	-1.375142514	2.941566398	k__Bacteria; p__Firmicutes; c__Clostridia; o__Clostridiales; f__Lachnospiraceae; g__Blautia; s__producta
aaba4784c870c05b77b3f1ab623babea	-1.569189489	3.134760072	k__Bacteria; p__Bacteroidetes; c__Bacteroidia; o__Bacteroidales; f__Bacteroidaceae; g__Bacteroides; s__coprophilus
d2952c12b62e658abe626d8491fbc315	0.391866744	3.226578166	k__Bacteria; p__Bacteroidetes; c__Bacteroidia; o__Bacteroidales; f__Bacteroidaceae; g__Bacteroides; s__
a3684b6011958f6e0cfe3f9073240352	-2.670077264	3.258207729	k__Bacteria; p__Firmicutes; c__Clostridia; o__Clostridiales; f__Lachnospiraceae
28ae94754fd30b89222bf9edcca2eb1f	-0.949958741	3.366639783	k__Bacteria; p__Fusobacteria; c__Fusobacteriia; o__Fusobacteriales; f__Fusobacteriaceae
88dbd9386e3162f377767f25035960de	-1.822579324	3.374314239	k__Bacteria; p__Bacteroidetes; c__Bacteroidia; o__Bacteroidales; f__[Paraprevotellaceae]; g__[Prevotella]; s__
297cfdbc22ba72f01af86d892d84a6d8	-1.18246597	3.385306051	k__Bacteria; p__Bacteroidetes; c__Bacteroidia; o__Bacteroidales; f__Bacteroidaceae; g__Bacteroides; s__

38bfbd3a2cf83da8fe03cea0f6b4f5f3	1.242392361	3.542211463	k__Bacteria; p__Proteobacteria; c__Gammaproteobacteria; o__Pseudomonadales; f__Pseudomonadaceae; g__Pseudomonas; s__fragi
83706970104da76b2528b23c82c19aef	-2.173661172	3.682051112	k__Bacteria; p__Firmicutes; c__Clostridia; o__Clostridiales; f__Lachnospiraceae; g__Blautia
5115f6aaca10f6ddcf3ffd4a89a05aaf	-0.182582319	4.013244083	k__Bacteria; p__Fusobacteria; c__Fusobacteriia; o__Fusobacteriales; f__Fusobacteriaceae
